# *SALL2*-Mediated Suppression of WNT Signaling Through Transcriptional Control of *AXIN2* in Colorectal Cancer Cells

**DOI:** 10.3390/ijms26167896

**Published:** 2025-08-15

**Authors:** Aracelly Quiroz, Emilia Escalona, Carlos Farkas, Diego Benítez-Riquelme, Paulina Sepúlveda, Mario Palma, Paula Medina, Carolina Delgado, Matías I. Hepp, Franz Villarroel-Espindola, Ariel F. Castro, Roxana Pincheira

**Affiliations:** 1 Departamento de Bioquímica y Biología Molecular, Facultad de Ciencias Biológicas, Universidad de Concepción, Concepción 4070409, Chile; aracellyquiroz@udec.cl (A.Q.); diegobenitez@udec.cl (D.B.-R.); psepulv.ps@gmail.com (P.S.); mpalma@hsph.harvard.edu (M.P.); pmedina2018@udec.cl (P.M.); 2 Laboratorio de Transducción de Señales y Cáncer, Facultad de Ciencias Biológicas, Universidad de Concepción, Concepción 4070409, Chile; 3 Departamento de Especialidades, Facultad de Medicina, Universidad de Concepción, Concepción 4070409, Chile; 4 MARLab, Instituto de Ciencias Biomédicas, Facultad de Ciencias de la Salud, Universidad Autónoma de Chile, Talca 3460000, Chile; emilia.escalona@uautonoma.cl; 5 Laboratorio de Investigación en Ciencias Biomédicas, Departamento de Ciencias Básicas y Morfología, Facultad de Medicina, Universidad Católica de la Santísima Concepción, Concepción 4030000, Chile; cfarkas@ucsc.cl (C.F.); mhepp@ucsc.cl (M.I.H.); 6 Translational Medicine Laboratory, Fundación Arturo López Pérez Cancer Center, Santiago 7500921, Chile; franz.villarroel@falp.org

**Keywords:** *SALL2*, *AXIN2*, Wnt-β catenin, CHIR99021, XAV393, CRC

## Abstract

Colorectal cancer (CRC) remains the second leading cause of cancer-related mortality worldwide, with aberrant activation of the Wnt/β-catenin signaling pathway constituting a key driver of tumorigenesis. *SALL2*, a zinc finger transcription factor deregulated in various cancers, has been implicated in Wnt signaling regulation through its Xenopus ortholog; however, its role in human CRC remains unclear. In this study, we investigated the expression and function of *SALL2* in CRC. Immunohistochemical analysis revealed that *SALL2* is present in the epithelium and stroma of normal colon tissue but is significantly downregulated in adenomas, carcinomas, and CRC cell lines. Reduced *SALL2* expression was associated with elevated levels of active β-catenin and poorer overall patient survival. Functional assays demonstrated that *SALL2* transcriptionally activates *AXIN2*, a key negative regulator of the Wnt/β-catenin pathway. Chromatin immunoprecipitation and promoter-reporter assays confirmed *SALL2* binding to the *AXIN2* proximal promoter and enhanced promoter activity. Furthermore, *SALL2* expression potentiated the pro-apoptotic effects of the Wnt pathway inhibitor XAV939 in CRC cells, suggesting a role in sensitizing cells to Wnt-targeted therapies. Collectively, these findings identify *SALL2* as a negative regulator of Wnt/β-catenin signaling and support its potential as a prognostic biomarker and therapeutic target in colorectal cancer.

## 1. Introduction

Colorectal cancer (CRC), by definition, includes carcinomas arising in the anatomical locations of the colon and rectum, and remains the second leading cause of cancer-related mortality worldwide [1,2]. Environmental factors and lifestyle choices are significant determinants of CRC development, as evidenced by the fact that most cases arise sporadically in patients without a family history of the disease [3]. CRC can be classified into four Consensus Molecular Subtypes (CMSs): CMS1, characterized by carcinomas with microsatellite instability and strong immune activation; CMS2, including canonical carcinomas with an epithelial phenotype; CMS3, comprising tumors with an epithelial phenotype and evident metabolic dysregulation; and CMS4, encompassing cancer with a stromal phenotype, prominent transforming growth factor-β (TGF-β) activation, high invasion, and angiogenesis [4]. Among these subtypes, CMS2 has the highest incidence (37%) and is characterized by hyperactivation of the Wnt/β-catenin pathway [5,6].

The canonical Wnt/β-catenin pathway is crucial for embryonic development and cell differentiation [7]. Activation of β-catenin depends on Wnt ligands binding to the Frizzled receptor and LRP co-receptor. This interaction disrupts the β-catenin destruction complex, which comprises *AXIN1/2*, APC, CK1, and GSK3β. In the absence of this complex, β-catenin accumulates in the cytosol and translocates into the nucleus, where it associates with TCF family transcription factors and co-activators to drive the transcription of target genes involved in cell proliferation, survival, cell adhesion, migration, stemness, and angiogenesis [8,9]. Multiple Wnt modulators and pharmacological approaches with therapeutic potential are currently in preclinical phases [10]. Therefore, identifying novel targets or genetic biomarkers is crucial for improving CRC therapies.

*SALL2*/Sall2 is a transcription factor member of the Spalt-like (SALL) family, conserved across many organisms, from nematodes to humans [11]. It has two main isoforms, E1 and E1A, which are generated by two alternative promoters, P1 and P2, respectively. These isoforms differ by only 25 amino acids in the N-terminal domain. The E1 isoform contains a nuclear localization sequence and a conserved repressor motif absent in E1A, suggesting distinct functions for each isoform. The E1 isoform is restricted to specific tissues, such as the thymus, testis, and colon, whereas E1A is widely expressed in tissues and is mainly related to cancer [11,12,13,14].

*SALL2* is involved in brain and eye development and is implicated in the regulation of key cellular processes, including cell proliferation, migration, and survival [11,15]. *SALL2* negatively regulates the cell cycle by repressing *CCDN1* and *CCNE1* expression and increasing *CDKN2A* and *CDKN1A* expression through transcriptional mechanisms [16,17,18]. Additionally, *SALL2* is a proapoptotic regulator that increases the expression of *BAX* and *PMAIP1* under genotoxic stress [19]. *SALL2* is mutated and/or downregulated in various cancers; however, its role has been investigated only in a few, such as ovarian, breast, and leukemia [12,15], confirming its tumor suppressor activity [16,19]. Interestingly, *SALL2* is upregulated in glioblastoma, and together with other neurodevelopmental factors, it promotes glioblastoma propagation [20], suggesting a dual role for *SALL2* in cancer.

Previous analyses of public datasets have shown a significant downregulation of *SALL2* mRNA expression in CRC samples compared to normal tissues [12], suggesting that its tumor suppressor activity is involved in colon carcinogenesis. Interestingly, the Xenopus sal (XsalF) ortholog negatively regulates the Wnt/β-catenin pathway by transcriptionally activating the negative regulators *tcf3* and *gsk3b*, resulting in the overall suppression of the pathway activity [21]. However, the relationship between *SALL2* and CRC, the role of the human ortholog in the Wnt/β-catenin pathway, and its influence on tumor progression and CRC molecular features remain unexplored.

Here, we aimed to investigate the role of *SALL2* in CRC by evaluating its protein expression levels in a cohort of human CRC samples and delving into the molecular mechanisms of *SALL2* function over the Wnt/β-catenin pathway using both loss and gain of function CRC cell models.

We found that *SALL2* is expressed in normal colon tissues but is significantly downregulated in adenoma and carcinoma samples, as well as cancer cell lines. Mechanistically, our study identified an inverse correlation between *SALL2* expression and nuclear β-catenin accumulation in both tissues and cell lines, suggesting that *SALL2* negatively regulates Wnt signaling. Furthermore, we demonstrated that *SALL2* transcriptionally upregulates *AXIN2* expression, a well-characterized negative regulator of the Wnt/β-catenin pathway. Finally, we showed that *SALL2* expression sensitizes CRC cells to death after treatment with a well-established Wnt signaling pathway inhibitor, highlighting the potential importance of *SALL2* in the effectiveness of future Wnt-targeted therapies for CRC.

## 2. Results

### 2.1. SALL2 Is Expressed in Normal Colon but Is Downregulated in Colorectal Cancer (CRC)

A previous report indicated that *SALL2-E1A* mRNA levels decreased in CRC samples [11]. In line with this, analyses of the UALCAN and GEPIA datasets revealed a significant reduction (*p* < 0.05) in *SALL2* mRNA levels in CRC tissues compared to normal tissues (Supplementary Figure S1a,b). However, *SALL2* protein expression has not been evaluated in CRC tissues. Therefore, we assessed *SALL2* expression through IHC on human CRC samples.

Our cohort consisted of 130 paraffin-embedded samples from CRC patients, which included 42 normal adjacent tissues, 40 adenomas, and 48 adenocarcinoma samples. Colorectal cancer is generally considered a single clinical and pathological entity encompassing both colon and rectal tumors. Although some molecular differences exist, the distinction is primarily anatomical and does not impact the overall classification or staging. According to the 9th edition of the TNM Classification [2] and major pathological guidelines (e.g., Pathology Outlines, https://www.pathologyoutlines.com/ and WHO, https://www.who.int, accessed on 17 April 2023), both tumor types are staged and categorized under colorectal adenocarcinoma. Our cohort included 4 rectal tumors among the 48 CRC cases analyzed. The average age of patients was 67.7 years, and the cohort included individuals of both sexes (Supplementary Table S1).

**Figure 1:**
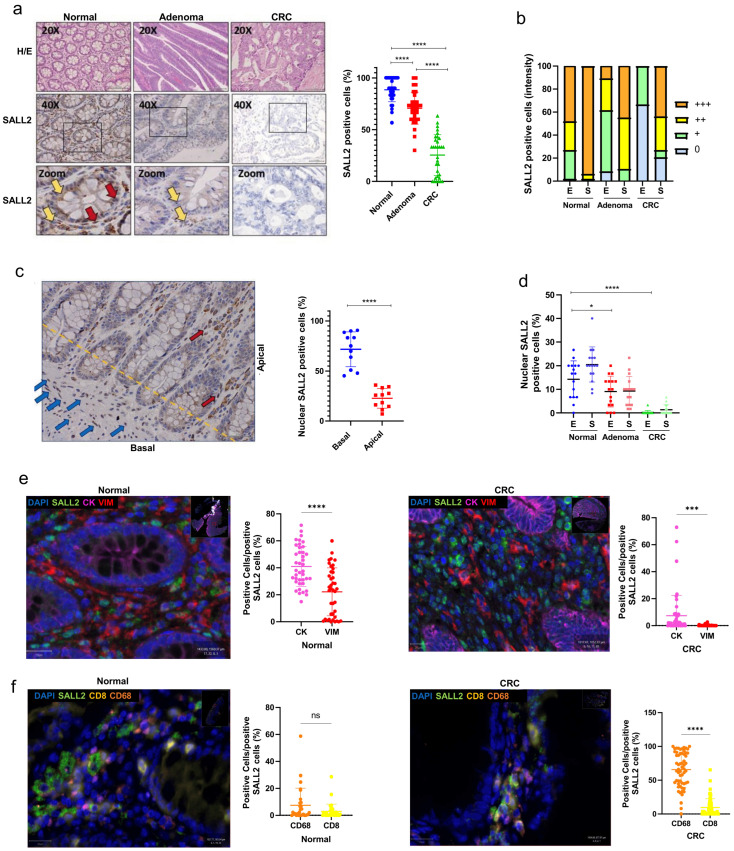
*SALL2* is expressed in both colon normal epithelial and stromal tissues but loses its expression in colorectal cancer (CRC). (**a**) Representative IHC images of *SALL2* in adenomas (*n* = 40), CRC (*n* = 48), and normal adjacent mucosa (*n* = 42) colon tissues (**left** panel). The third line of figures provides a close-up view of the section located in the center of the black box region. Scale bars: 200 μm. Percentage of *SALL2*-positive cells from the IHC score (**right** panel). Yellow arrows indicate *SALL2* cytosolic expression. Red arrows indicate *SALL2* nuclear expression (**b**). The *SALL2* intensity score (mean) decreases from normal to CRC progression. The *SALL2* intensity was graduated as negative: 0, weak: +, moderate: ++, and strong: +++. From each group, *SALL2* intensity was evaluated in the epithelium (E) and stroma (S). (**c**) Representative IHC image showing the distribution of *SALL2* in the colon crypt. Percentage of nuclear *SALL2*-positive cells in the basal and apical regions (right panel). The blue arrows indicate the presence of *SALL2* expression at the crypt’s base, while the red arrows highlight its expression in the gland’s apical region. (**d**) Percentage of nuclear *SALL2*-positive cells in the epithelium (E) and stroma (S) from normal to CRC tissues. (**e**,**f**) Representative images of *SALL2* staining in the stroma of the normal colon tissue (**left**) and the tumor microenvironment in CRC tissues (right) using multiplex immunofluorescence analysis. Quantification is shown alongside each image using automated color-based threshold segmentation in the free software QuPat v0.6.0. Regions of interest (ROIs) were manually selected, distinguishing between normal and cancerous areas. The graphs show the percentage (%) of cells positive for *SALL2* and the specific markers. Each plotted point represents a single ROI. The immunofluorescence *SALL2* (green), fibroblasts (VIM, vimentin in red), and epithelium (CK, cytokeratin in magenta) are shown in (**e**). The *SALL2* (green), cytotoxic lymphocytes (CD8+ in yellow), and macrophages (CD68+ in orange) are shown in (**f**). All data are mean ± SD. Proportional and intensity scores were quantified as described in the Material and Methods Section. Statistical significance was determined by ANOVA (**** *p* < 0.0001; *** *p* < 0.001; * *p* < 0.05, ns, not significant).

The percentage of *SALL2*-positive cells was 90.6% in adjacent normal tissues, 74.3% in adenomas, and 25.4% in CRC tissues (Figure 1a). Analysis of *SALL2* intensity revealed that *SALL2* is expressed in both the stroma and epithelium of the normal colon, with higher abundance in the stromal compartment (Figure 1b). Additionally, *SALL2* was localized in the crypts of the colonic epithelium, where it was notably concentrated in the basal regions, particularly within Lieberkühn’s crypts associated with proliferative niches (Figure 1c). We evaluated the nuclear localization of *SALL2* because its function depends on this subcellular localization [11]. In adjacent normal tissue, nuclear *SALL2* staining was more prominent in stromal cells (23.3%) compared to in epithelial cells (or glands, 15.4%) (indicated by yellow arrows in Figure 1a). However, there was a significant decrease in nuclear *SALL2* staining in both cell types as the tissue progressed from normal to adenoma and CRC (Figure 1d). In summary, *SALL2* protein levels were significantly reduced in adenomas and adenocarcinoma tissues (*p* < 0.01) compared to in adjacent normal tissues.

We then examined the specific stromal cell types that express *SALL2*. We generated two triple-staining panels: one labeling *SALL2*, vimentin (a fibroblast marker), and cytokeratin (an epithelial cell or enterocyte marker), and the other labeling *SALL2*, CD8 (a cytotoxic lymphocyte marker), and CD68 (a macrophage marker) to identify immune cells. IHC analysis using QuPath software revealed that 40.3% of cytokeratin-positive cells in normal tissue were also *SALL2*-positive, compared to just 7.5% in cancer tissue. Similarly, 21.8% of vimentin-positive cells were *SALL2*-positive in normal tissue, whereas only 1% in cancer tissue showed this positivity (Figure 1e). These findings indicated that *SALL2* is primarily localized in epithelial enterocytes and fibroblast cells in normal colon tissue. From panel two, *SALL2*-CD8-positive cells were sparse (less than 10%) in both normal and cancer tissues. In contrast, *SALL2*-CD68-positive cells were significantly more abundant in CRC (65.7%) compared to normal tissue (7.5%) (Figure 1f).

*SALL2* has been characterized as a tumor suppressor in various tissues, including the ovary and breast [13,14]. Loss of *SALL2* expression in CRC may correlate with a worse patient prognosis. Notably, data from public data sets provided by R2 Genomics show that patients with the lowest levels of *SALL2* in CRC tissues have worse survival outcomes than those with higher *SALL2* expression. Our local study showed a similar trend (Supplementary Figure S1c,d).

In summary, we presented evidence that *SALL2* is predominantly expressed in the stroma and epithelium of the colon and is significantly decreased in CRC tissues. These results and the association between lower *SALL2* expression and poor patient survival support the notion of *SALL2* functioning as a tumor suppressor in CRC.

### 2.2. The Loss of SALL2 Is Associated with an Increase in Nuclear β-Catenin in CRC Cells and Tissue Samples

**Figure 2:**
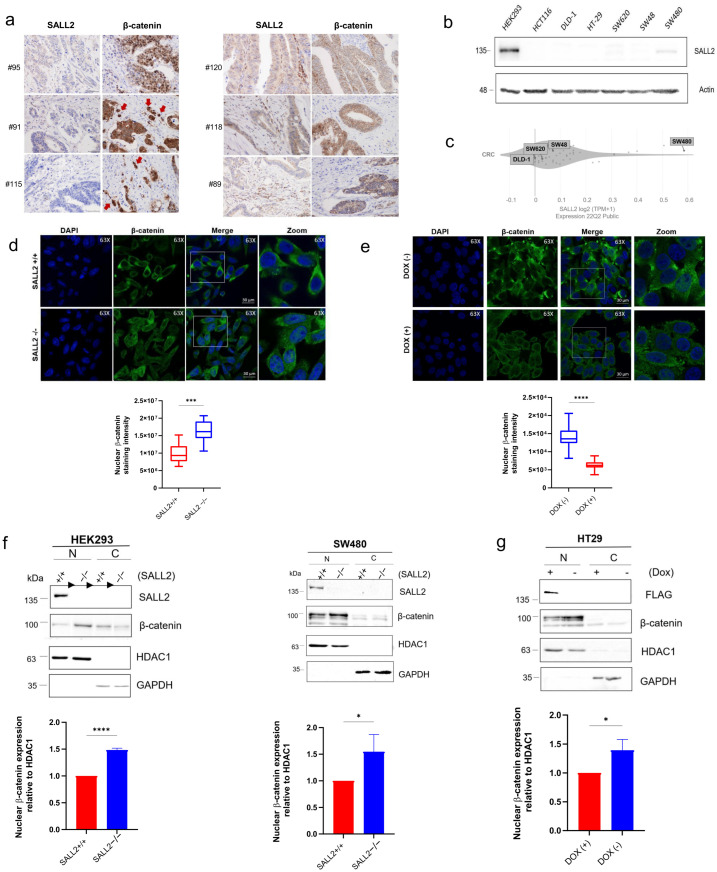
*SALL2* expression negatively correlates with the nuclear β-catenin cancer marker. (**a**) Immunohistochemical analysis of *SALL2* and β-catenin protein expression in colorectal cancer (CRC) tissues. Left, Representative images from 3 patients with negative *SALL2* and positive β-catenin (nuclear) staining at the migratory front (arrows). Right, Representative images from 3 patients with positive *SALL2* and negative β-catenin (membranous) staining. β-catenin staining was analyzed considering localization and intensity. (**b**) Western blot analysis of *SALL2* expression in CRC cell lines and HEK293 cells as a positive control. β-actin was used as a loading control. (**c**) Depmap analysis of *SALL2* mRNA expression in CRC cell lines (https://depmap.org/portal/ accessed on 7 July 2023). (**d**) Immunofluorescence analysis by confocal microscopy for endogenous β-catenin expression and localization in the *SALL2*+/+ and *SALL2*−/− SW480 cells. Quantifying nuclear β-catenin staining (green) intensity was performed using Fiji software, analyzing 100 cells across three independent experiments (Bottom panel). (**e**) Same as (**d**), but for the *SALL2* gain-of-function SW480 cells treated with vehicle [DOX(−)] or doxycycline [DOX(+)] to induce *SALL2* expression. (**f**) Western blot analysis of β-catenin expression in nuclear (N) and cytosolic (C) fractions from *SALL2*+/+ and *SALL2*−/−HEK293 (**Left**) and SW480 (**Right**) cell models. Nuclear levels of β-catenin were normalized to HDAC1 and expressed as a percentage relative to the control. Endogenous *SALL2* expression is shown. GAPDH was used as a control for the cytosolic fraction. The arrowheads indicate cropped unrelated columns and subsequent splicing of the blot. (**g**) Same as (**f**), but for the *SALL2* gain of function HT29 CRC cell model treated with vehicle (−) or doxycycline (+) to induce *SALL2* expression, detected with FLAG antibody. GAPDH and HDAC1 were used as controls for the cytosolic and the nuclear fractions, respectively. All data are mean ± SD. Statistical significance was determined by Student’s *t*-test (**** *p* < 0.0001, *** *p* < 0.001, * *p* < 0.05).

Beta-catenin is a key component of the Wnt signaling pathway, acting as a master regulator of cell motility and playing a crucial role in colon cancer invasion and progression [8,9]. The abnormal accumulation of nuclear β-catenin and a mixed staining pattern encompassing nuclear and cytoplasmic localization are positive markers for CRC progression [22,23]. Conversely, when β-catenin is exclusively located at the cell membrane, it indicates negative cancer progression and is associated with the inactivation of the Wnt pathway. Therefore, to evaluate the association between *SALL2* expression and Wnt pathway activity in clinical samples, we used the β-catenin staining pattern as a marker. We found a negative association between *SALL2* and nuclear β-catenin staining. In cases where *SALL2* showed negative staining, β-catenin was positive at the migratory front in 80% of patients (Supplementary Table S2). *SALL2*-negative CRC samples correlated with strong nuclear β-catenin staining at the migratory front and positive lymphovascular permeation (Figure 2a, left). In contrast, *SALL2*-positive CRC samples correlated with β-catenin localized at the plasma membrane and negative lymphovascular permeation (representative images are shown in Figure 2a, right).

These findings were also related to tumor invasion to the serosal surface; in the absence of *SALL2*, 75.61% of CRC tissues exhibited higher invasion than the *SALL2*-positive tissues (24.39%) (Supplementary Table S2). Otherwise, the expression of *SALL2* did not correlate with microsatellite stability or proliferation (Supplementary Table S2). Our analysis indicated that the loss of *SALL2* during CRC progression correlates with active β-catenin, suggesting that *SALL2* regulates the Wnt pathway.

Next, we conducted Western blot analysis to evaluate *SALL2* expression in various CRC cell lines. Using lysates from HEK293 cells as a positive control for *SALL2* expression [24], we observed a marked reduction in *SALL2* levels in most CRC cell lines tested (Figure 2b), with detectable expression observed only in SW480 cells. These results were consistent with a Depmap RNA analysis of CRC cells, where *SALL2* mRNA was expressed in SW480 but not DLD-1, SW48, and SW620 cell lines (Figure 2c).

To better understand the *SALL2*−β-catenin relationship, we used *SALL2* loss-of-function and gain-of-function CRC models. Confocal microscopy analysis showed an increased nuclear distribution of endogenous β-catenin in *SALL2*KO (*SALL2*−/−) SW480 cells compared to that of *SALL2*+/+ SW480 cells (Figure 2d). This phenotype was reversed by doxycycline-induced expression of *SALL2* in the *SALL2*−/− cells (Figure 2e). These results were confirmed by cell fractionation and Western blot analysis. In addition to the SW480 cells, we used HEK293 cells because they have an intact Wnt network, respond to canonical Wnt ligands [25,26] and express high levels of *SALL2* [24]. We extracted the cytoplasmic and nuclear proteins from the *SALL2* wild type (*SALL2*+/+) and deficient (*SALL2*−/−) models (HEK293 and SW480) and evaluated β-catenin expression by Western blot. *SALL2*−/− cells showed significantly higher levels of nuclear β-catenin than the *SALL2*+/+ models (Figure 2f). Conversely, the gain of *SALL2* expression in another CRC cell model (doxycycline-inducible HT29 cells) significantly decreased nuclear β-catenin levels (Figure 2g).

These results suggest that *SALL2* expression attenuates the nuclear accumulation of β-catenin in colon tissues and cells, indicating that *SALL2* restrains the Wnt/β-catenin signaling pathway.

### 2.3. SALL2 Is an Antagonistic Mediator of Wnt/β-Catenin Signaling in CRC Cells

**Figure 3:**
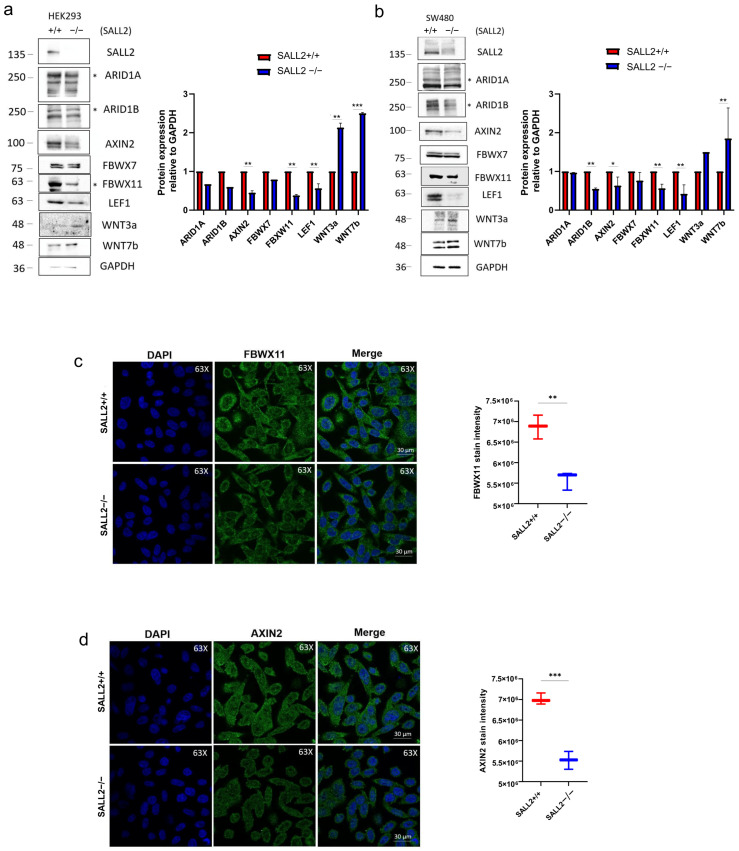
Loss of *SALL2* function downregulates negative regulators of the Wnt Pathway. (**a**) Left, Western blot analysis of the Wnt pathway negative and positive regulators expression in *SALL2*+/+ and *SALL2*−/− HEK293 cells. (**Right**) Densitometric analysis of the bands from 3 independent experiments is shown on the left. Protein levels were normalized to GAPDH, a loading control. The “*” on the right of the blot indicates specific bands. (**b**) Same as (**a**), but for *SALL2*+/+ and *SALL2*−/− SW480 cells. (**c**) (**Left**) Immunofluorescence analysis by confocal microscopy for FBWX11 expression in *SALL2*+/+ and *SALL2*−/− SW480 cells. Right, Quantification of FBWX11 staining intensity was performed using Fiji software, analyzing 100 cells across three independent experiments. (**d**) Same as (**c**), but for *AXIN2* expression. All data are mean ± SD. Statistical significance was determined by Student’s *t*-test (*** *p* < 0.001; ** *p* < 0.01; * *p* < 0.05).

To investigate how *SALL2* modulates the Wnt pathway, we assessed the expression changes of both negative and positive regulators of the Wnt/β-catenin pathway in our cell models using Western blot analysis. In *SALL2*−/− cells, we observed a significant increase in the levels of the Wnt agonists WNT3A and WNT7B, alongside a notable decrease in the levels of two negative Wnt/β-catenin pathway regulators, *AXIN2* and FBXW11, compared to the *SALL2* +/+ cells (Figure 3a,b).

Given the important roles of FBXW11 and *AXIN2* in regulating cytoplasmic β-catenin stability [27,28], we further analyzed their expression and subcellular localization using confocal microscopy. Consistent with the Western blot results, the loss of *SALL2* significantly decreased the signal intensity of both cytoplasmic *AXIN2* (Figure 3c) and FBXW11 (Figure 3d). However, the subcellular localization of these proteins seemed to be independent of *SALL2*.

Additionally, we conducted a differential expression analysis to investigate the association between *SALL2* and the Wnt pathway-related genes in cancer. This analysis, conducted in 14 major cancers using the web tool TIMER 2.0 confirmed our previous observations. Specifically, cancers with *SALL2* mutations displayed upregulated *WNT7B* and downregulated *AXIN2* (Supplementary Figure S2). The findings indicate that loss of *SALL2* in CRC cells is linked to changes in the expression of important Wnt pathway regulators, implying a connection between *SALL2* and the suppression of the Wnt/β-catenin pathway.

### 2.4. The Expression of the Wnt/β-Catenin Regulator AXIN2 Depends on SALL2 Expression

**Figure 4:**
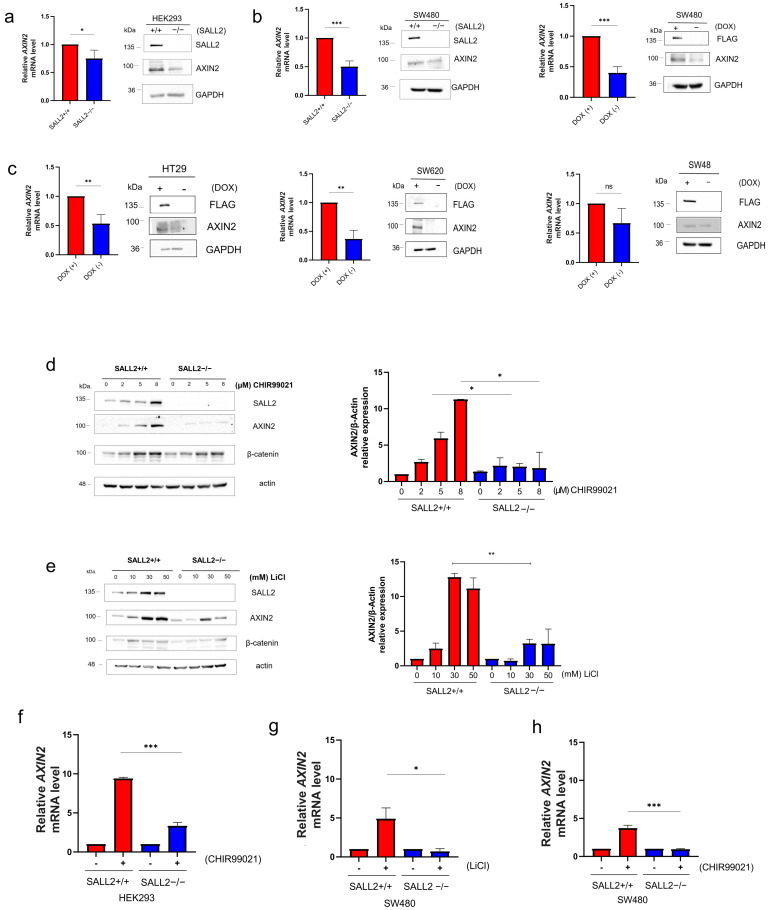
Transcriptional regulation of *AXIN2* depends on *SALL2* expression. (**a**) *AXIN2* mRNA levels and protein were analyzed by qPCR and Western blot, respectively, in *SALL2* +/+ and *SALL2*−/− HEK293 cell models. (**b**) Left, Same as (**a**), but for the *SALL2*+/+ and *SALL2*−/− SW480 cell models. (**Right**) Same as (**a**), but for inducible FLAG-*SALL2* E1A SW480 cells treated with 1 µg/mL doxycycline (DOX) for 48 h. (**c**) The same analyses were performed on inducible FLAG-*SALL2* E1A HT-29, SW620, and SW48 CRC cells. qPCR and immunoblotting were conducted using *PP1B* and *GAPDH* as normalization controls, respectively. (**d**,**e**) Western blot analysis of *AXIN2* and *SALL2* expression in response to 4 h treatment with different concentrations of the Wnt pathway activators CHIR99021 (**d**) and LiCl (**e**) in *SALL2* +/+ and *SALL2*−/− HEK293 cells. β-catenin was used as a positive control for the Wnt pathway activation. Graphs on the right show the quantification of *AXIN2* levels relative to β-actin (loading control) by densitometry analysis of Western blots. (**f**) The *SALL2*+/+ and *SALL2*−/− HEK293 cells were treated with 8 µM CHIR99021 for 4 h and the *AXIN2* mRNA expression relative to *PPIB* was analyzed by qPCR. *AXIN2* mRNA expression relative to *PPIB* was analyzed by qPCR in the *SALL2*+/+ and *SALL2*−/− SW480 cells treated with 10 mM LiCl for 4 h (**g**) or 8 µM CHIR99021 for 4 h (**h**). All data are mean ± SD. Statistical significance was determined by ANOVA or Student’s *t*-test (*** *p* < 0.001; ** *p* < 0.01; * *p* < 0.05; ns, not significant).

We focused our analysis on understanding the *SALL2*-*AXIN2* relationship. We analyzed whether *AXIN2* mRNA expression depends on *SALL2* using non-tumor HEK293 and several CRC cell models. Loss of *SALL2* in HEK293 cells led to a significant decrease in *AXIN2* mRNA levels compared to those in the *SALL2*+/+ condition (Figure 4a, left). This decrease in *AXIN2* mRNA expression was correlated with a decrease in *AXIN2* protein expression (Figure 4a, right). As SW480 cells were the only CRC cell line with detectable *SALL2* expression, we investigated how the loss of *SALL2* affects *AXIN2* mRNA levels in CRC cells. Similar to the HEK293 cells, loss of *SALL2* was correlated with a decrease in *AXIN2* mRNA and protein levels (Figure 4b, left). This decrease in *AXIN2* expression was reversed by reintroducing *SALL2*, confirming the rescue of *AXIN2* regulation (Figure 4b, right).

We further investigated the *SALL2*-*AXIN2* relationship in other CRC cell models. As *SALL2* expression was undetectable in HT29, SW620, and SW48 CRC cells, we analyzed how inducible knock-in of *SALL2* affected *AXIN2* mRNA in these cells. Rescue of *SALL2* expression significantly increased *AXIN2* mRNA and protein levels in HT29 and SW620 CRC cells (Figure 4c, left and middle). Although a similar trend was observed in SW48 CRC cells, the increase in *AXIN2* expression was not significant (Figure 4c, right).

Because *AXIN2* is a direct target of the Wnt pathway, regulated by TCF/LEF factors [28], we examined the function of *SALL2* in the context of two established activators of the canonical Wnt signaling pathway: lithium chloride (LiCl) and CHIR99021. These compounds inhibit GSK3β activity and stabilize cytosolic β-catenin [29,30]. Nevertheless, due to LiCl’s potential to influence additional pathways, our primary focus was on CHIR99021, which serves as a more targeted inhibitor of GSK3 [30,31,32]. *AXIN2* expression relied on *SALL2* under the Wnt pathway activation in HEK293 cells (Figure 4d,e). In the presence of both Wnt agonists, *AXIN2* expression increased dose-dependently. This effect was abolished in the *SALL2*−/− HEK293 cells (Figure 4d,e). The qPCR analysis confirmed that *AXIN2* mRNA induction depended on *SALL2* and CHIR99021 (Figure 4f). Similarly, both agonists significantly increased *AXIN2* mRNA in the *SALL2*+/+ SW480 cells but not in the *SALL2*−/− SW480 cells (Figure 4g,h), indicating that *SALL2* is required for *AXIN2* transcription, even under the Wnt pathway activation.

Altogether, our results strongly suggest that *SALL2* is a novel positive regulator of *AXIN2* gene transcription in the absence and presence of Wnt agonists.

### 2.5. SALL2 Attenuates Wnt/β-Catenin Signaling by Directly Transactivating the AXIN2 Promoter in CRC Cells

**Figure 5:**
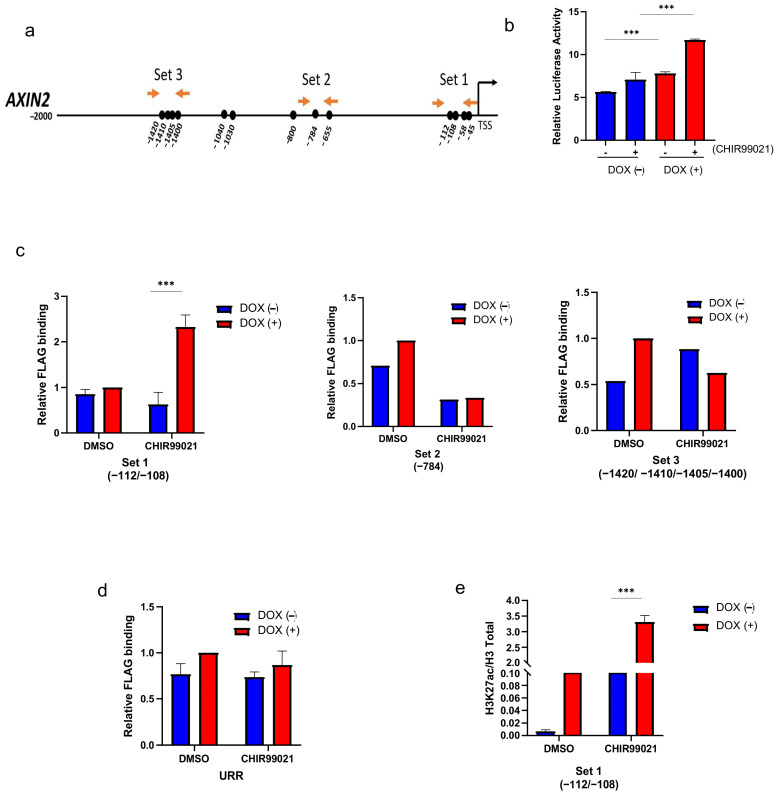
*SALL2* binds to the *AXIN2* promoter. (**a**) Schematic representation of the human *AXIN2* promoter. Bioinformatic analysis of the promoter to identify putative *SALL2* sites was performed using a previously reported binding site matrix (consensus sequence GGG(T/C)GGG) [34]. The human *AXIN2* sequence analyzed (2000 bp) from the transcription start site (TSS) was obtained from the Eukaryotic Promoter Database (EPD) (https://epd.expasy.org/epd/ accessed on 12 September 2023). Circles are schematic representations of *SALL2* putative binding sites. Horizontal arrows indicate the location of the primers used for qPCR (sets 1, 2, and 3) in site-specific ChIP assays. (**b**) *AXIN2* promoter activity was measured in the absence (DOX (−)) and presence of *SALL2* (DOX (+)) in HEK293 cells treated with 8 µM CHIR99021 for 4 h. Luciferase activity was measured from cell lysates and normalized to β-galactosidase activity, and promoter activity was expressed as relative luciferase activity. (**c**) Analysis of *SALL2* enrichment in the *AXIN2* promoter. Chromatin from doxycycline-induced FLAG-*SALL2* E1A HEK293 cells was immunoprecipitated using FLAG antibody. Specific genomic regions of the *AXIN2* promoter were analyzed by qPCR. The *AXIN2* promoter was analyzed using 3 primer sets, set 1 (−08/−112), set 2 (−784), and set 3 (−1420/−1410/−1405/−1400). (**d**) Analysis of *SALL2* enrichment in an unrelated region (URR) corresponding to the *PMAIP1* gene promoter. (**e**) H3K27 acetylation enrichment relative to total histone H3. All data are mean ± SD. Statistical significance was determined by ANOVA test (*** *p* < 0.001).

To further investigate the *SALL2*-dependent transcriptional regulation of *AXIN2*, we conducted a bioinformatics analysis of *SALL2* binding sites within the 2 kb regions of the human *AXIN2* promoter (ENSG00000168646). We used a previously reported binding site matrix [33,34]. Interestingly, we identified thirteen putative *SALL2* binding sites in the *AXIN2* promoter, as illustrated in Figure 5a. Seven of these sites were located within the proximal promoter region.

Next, through a luciferase-reporter assay, we evaluated the responsiveness of a previously reported human 1 Kb *AXIN2* promoter [35], which contains 7 out of 13 *SALL2* putative sites. We focused on the *SALL2* E1A isoform because it exhibits the most significant overall change in expression levels in cancer [12]. Using the *SALL2*−/− HEK293 cells, we generated the *SALL2E1A* doxycycline-inducible HEK293 model. Cells were cultured in the absence and presence of doxycycline for 24 h, followed by treatment with CHIR99021 for 4 h. *SALL2* E1A significantly increased the basal and the Wnt pathway-dependent *AXIN2* promoter activity (Figure 5b), indicating that the *SALL2* E1A isoform positively regulates the *AXIN2* promoter activity.

To demonstrate the in vivo interaction of *SALL2* E1A with the *AXIN2* promoter, we performed a chromatin immunoprecipitation (ChIP) assay using the inducible *SALL2E1A*-HEK293 cells in the absence and presence of doxycycline and CHIR99021. Three specific primer sets (1 to 3) were used to evaluate *SALL2* binding to different regions of the *AXIN2* promoter (Figure 5a).

*SALL2* E1A was bound to the proximal region of the *AXIN2* promoter (-108/-112 from TSS), amplified by primers set 1, and its binding increased under CHIR99021 treatment; *SALL2* binding was not observed in other distant regions (set 2 and set 3) (Figure 5c). In contrast, no *SALL2* binding was detected in a non-related region [33] (URR; Figure 5d). The increase in *SALL2* binding to the proximal region (set 1) after CHIR99021 treatment was correlated with a significant increase in histone H3 acetylation (H3K27ac) (Figure 5e), which is a marker of transcriptional activation [36,37]. These results demonstrated that *SALL2* E1A binds and activates the *AXIN2* promoter.

### 2.6. Suppression of Wnt Signaling by XAV939 Increased CRC Apoptosis in a SALL2-Dependent Manner

Tankyrase 1 inhibitor XAV939 is a small molecule inhibitor of the Wnt signaling pathway; it blocks Wnt signaling by stabilizing the *AXIN2* protein, which leads to increased β-catenin destruction [38,39]. It was shown that *AXIN2* gene silencing in embryonic stem cells (ESCs) reduces apoptosis by regulating the mitochondria-associated apoptosis pathway and modulating the Wnt/β-catenin signaling [40]. Consistent with the association of *AXIN2* with apoptosis, the XAV939 inhibitor has been reported to significantly enhance apoptosis in SW480 cells, along with an increase in *AXIN2* protein levels [41].

**Figure 6:**
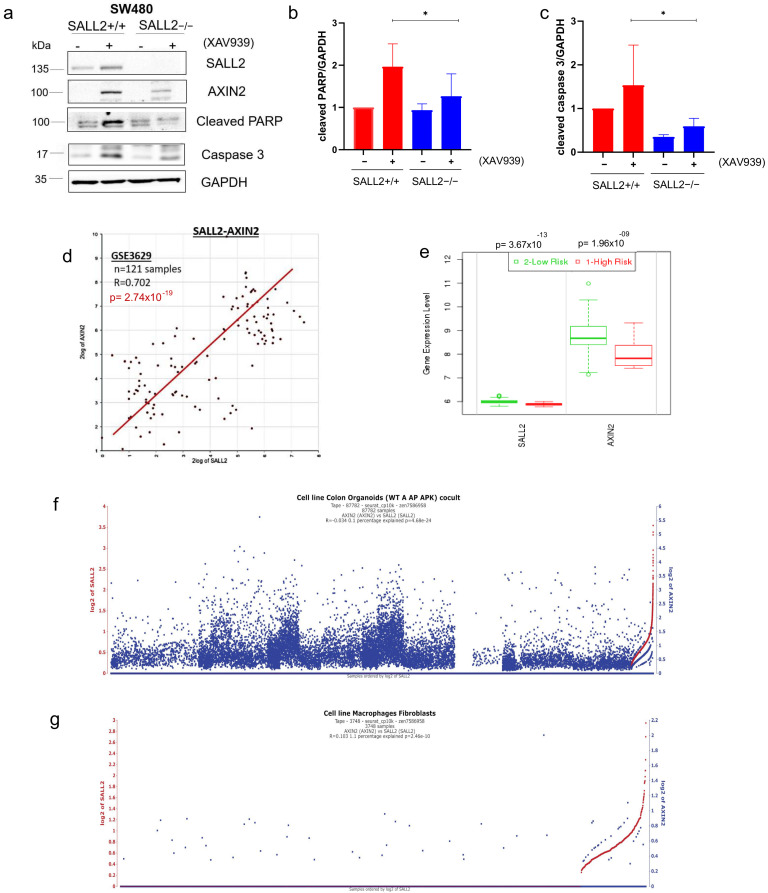
*SALL2* expression increases XAV939-induced cellular apoptotic response and is associated with *AXIN2* expression and better prognosis in colorectal cancer (CRC). (**a**) *SALL2*+/+ and *SALL2*−/− SW480 cells were treated with 8 µM XAV939 for 24 h. The apoptotic response was evaluated by the levels of apoptotic markers: cleaved caspase 3, and cleaved PARP. AXIN 2 was used as a positive control for the XAV939 treatment. GAPDH was used as a loading control. (**b**) Histogram of densitometric analysis for cleaved PARP expression from 3 independent experiments. (**c**) Histogram of densitometric analysis for cleaved caspase 3 expression from panel A. All data are mean ± SD. Statistical significance was determined by ANOVA test (* *p* < 0.05). (**d**) Correlation between *SALL2* and *AXIN2* expression in CRC. Scatter plots of *SALL2* vs. *AXIN2* were generated using publicly available databases and software from R2: Genomics Analysis and Visualization Platform (http://r2.amc.nl, accessed on 15 November 2023), *n* = 121 samples, R = 0.702, *p*-value = 2.7 × 10^−19^. (**e**) Same analysis as (**d**) for the Single-cell RNA-seq dataset from Qin et al. (87 782 cells) showing *SALL2*–*AXIN2* co-expression across all cell types. (**f**) Same analysis restricted to the macrophage/fibroblast subset of the Qin dataset confirms the positive correlation (R = 0.103, *p*-value = 2.46 × 10^−10^). (**g**) Boxplot across CRC risk groups and *AXIN2* or *SALL2* correlation, including the *p*-value testing for difference using Student’s *t*-test (or f-test for more than two groups) from the SurvExpress database. Green denotes high expression, while red denotes low expression.

Therefore, we investigated the role of *SALL2* in the XAV939-mediated apoptosis of CRC cells. Initially, *SALL2*+/+ SW480 cells were treated with XAV939 at various times to determine the optimal treatment conditions. *AXIN2* and β-catenin were used as controls to assess the effects of the inhibitor. As expected, *AXIN2* protein levels increased after 12 h, reaching maximum accumulation at 24 h; consequently, β-catenin levels significantly decreased at that time. Interestingly, *SALL2* levels also increased at 24 h (Figure 6a).

Next, we evaluated the expression of apoptotic markers in the *SALL2*+/+ and *SALL2*−/− SW480 cells treated with XAV939 for 24 h. Consistent with a previous report, XAV939 treatment increased cellular apoptosis, as evidenced by the marked increase in cleaved caspase 3 and PARP (Figure 6a–c). However, levels of *AXIN2* and the apoptotic markers were significantly diminished in the *SALL2*−/− cells (Figure 6a). These results suggest that the loss of *SALL2* is associated with resistance to the Wnt pathway inhibition and cell death induced by XAV939.

Finally, we analyzed the potential clinical significance of the association between *SALL2* and *AXIN2*. Further reinforcing their association, we found a positive correlation (R = 0.702, *p* = 2.74 × 10^−19^) in a colon cancer study (GSE3629) using R2 analysis (Figure 6d). Additionally, we examined a single-cell RNAseq analysis of CRC tumor microenvironment organoids [wild-type (WT), shApc (A), shApc and KrasG12D/+ (AK), shApc, KrasG12D/+, and Trp53R172H (AKP)] [42]. This analysis showed that a subset of *AXIN2* positive/*SALL2*-positive cells in the tumor organoids microenvironment depicts a strong positive correlation between the two genes. Notice the steep correlation curve at the end between *AXIN2* expression and *SALL2*-positive cells, regardless of global correlation (Figure 6e,f). Both studies demonstrate a positive correlation between *SALL2* and *AXIN2* in CRC. Moreover, employing the SurvExpress platform, we found evidence that colon cancer patients with higher cancer risk exhibited lower levels of *SALL2* and *AXIN2* compared to those with lower cancer risk (Figure 6g). However, data from primary CRC tumors (TCGA) and CBioportal web [43] showed a negative association between *SALL2* and *AXIN2* mRNA levels (Supplementary Figure S3), suggesting that the effect of *SALL2* on *AXIN2* might be context-dependent.

In summary, we showed that *SALL2* is required for the sensitivity of CRC cells to the Wnt pathway inhibition. We also found evidence of a positive association between *SALL2* and *AXIN2* in CRC and that their loss is associated with higher CRC risk. Overall, we suggest that the *SALL2*-*AXIN2* axis is an essential novel component of the Wnt/β-catenin signaling pathway and a potential molecular target for reversing drug resistance in colon cancer.

## 3. Discussion

*SALL2* is a transcription factor that plays key roles in embryonic development, cell growth, programmed cell death, and cancer progression. It is regarded as a potential tumor suppressor because of its ability to inhibit cell proliferation and promote apoptosis in response to genotoxic stress [11]. *SALL2* regulates the expression of tumor suppressor genes, including *CDKN1A*, *CDKN2A*, *PMAP1*, and *PTEN*, while repressing oncogenes such as *CCND1*, *CCNE1*, and *c-MYC* [14,16,18,19,44]. Studies have shown that *SALL2* mRNA levels are diminished in several types of cancer, with a particularly significant reduction in the *SALL2E1A* isoform mRNA in colon cancer [12,13,14]. However, no functional studies have been conducted on *SALL2* in colorectal cancer.

This study is the first to examine *SALL2* protein expression and subcellular localization in CRC. Previous studies have shown that the expression and localization of *SALL2* vary among different tissue types in a context-dependent manner. *SALL2* is primarily nuclear in normal ovarian tissue but becomes undetectable in ovarian cancer. In breast cancer, it displays both nuclear and cytoplasmic patterns in primary tumors, but its expression disappears in advanced metastatic stages [14]. Our findings revealed that *SALL2* is present in the normal colonic mucosa in both the nucleus and cytoplasm, with a predominant presence in the cytoplasm. Notably, *SALL2* expression was significantly decreased in neoplastic tissues. We also observed higher levels of nuclear *SALL2* expression in the stroma than in normal glands. *SALL2* might be latent in the cytoplasm, as reported in neuronal cells [45], whereas nuclear *SALL2* in stromal cells could play a role in maintaining tissue homeostasis. Consistent with our findings, a recent large-scale bioinformatic study of cancer showed a positive correlation between *SALL2* and the stromal score in colon adenocarcinoma (COAD), stomach adenocarcinoma (STAD), and rectal adenocarcinoma (READ) [46]. Our multiplex IHC analysis indicated that *SALL2*-positive cells include epithelial enterocytes and fibroblasts in normal tissue. However, in colon adenocarcinoma, *SALL2* expression decreased in both cell types and increased in type 1 macrophages (CD68+). The p53 tumor suppressor controls the tumor microenvironment (TME) through various mechanisms, primarily by regulating the cellular secretome [47]. Like wild-type p53, we hypothesize that a decrease in *SALL2* levels in cancer-associated fibroblasts might influence the cellular secretome and, consequently, the tumor microenvironment. Given the essential role of the stroma in both normal colon epithelium and cancer progression, further investigation into the function of the *SALL2* transcription factor in stromal cells is necessary.

When investigating the Wnt/β-catenin signaling pathway, it is essential to evaluate the nuclear concentrations of β-catenin, as these levels correlate with the pathway’s transcriptional activity and serve as a prognostic indicator of cancer development [48]. Our data from human tissues and CRC cells revealed an inverse correlation between *SALL2* protein expression and the Wnt/β-catenin pathway activity. We demonstrated that *SALL2* positively regulates *AXIN2*, an inhibitor of the Wnt pathway, and loss of *SALL2* is associated with increased nuclear β-catenin levels. Additionally, *SALL2* may negatively regulate the Wnt3A and Wnt7B activators and positively regulate the Wnt pathway inhibitor FBXW11. Although further studies are necessary, these findings support the notion that *SALL2* functions as a novel regulator of the Wnt/β-catenin pathway. Interestingly, Onai et al. found that the Xenopus *SALL2* orthologue, XsalF, negatively affects the Wnt/β-catenin pathway by promoting the transcription of pathway inhibitors like *tcf3* and *gsk3-β* [21], suggesting a conserved regulatory mechanism of the Wnt pathway by SALLs.

ChIP experiments demonstrated that *SALL2* binds to the *AXIN2* proximal promoter in vivo; however, it remains uncertain whether this binding occurs directly to the consensus sequence or indirectly, as seen with its paralog SALL1. SALL1 influences the canonical Wnt pathway without directly binding to chromatin; instead, it interacts with β-catenin and, along with pathway activators such as Wnt3A, enhances Wnt signaling as a co-regulator [49]. Similarly, SALL4 upregulates the Wnt/β-catenin pathway activity by directly binding to and transactivating the *CTNNB1* gene promoter [50]. Regardless of the mechanism, *SALL2* functions in opposition to SALL1 and SALL4 in the Wnt/β-catenin pathway. Intriguingly, SALL1 is downregulated in colon cancer [51], whereas SALL4 is overexpressed in it [52]. These observations suggest a potential interplay between SALL family members and the Wnt signaling pathway in colorectal cancer.

Consistent with *SALL2*’s role in the Wnt pathway, similar to other negative regulators, such as *AXIN2* [53], activating this pathway with CHIR99021 or LiCl increases *SALL2* protein expression, which is likely necessary to control the level of pathway activation. The *SALL2* promoter contains TCF-LEF binding sites, which may explain transcriptional regulation [54]. Nonetheless, *SALL2* post-translational regulation may be involved. We recently demonstrated the regulation of *SALL2* stability by phosphorylation and proteasome-induced degradation via CK2. Treatment of SW480 CRC cells with CX4549, a CK2 inhibitor, increased *SALL2* protein levels and function [24]. Similarly, a post-translational regulation mediated by kinases of the Wnt pathway such as GSK3β could also exist, considering that both CHI99021 and LiCl inhibit GSK3 activity. Similarly, we observed that the inhibition of tankyrase with XAV939 increased *SALL2* protein levels.

Previous studies have shown that XAV939 induces apoptosis in the SW480 cell model, making it more susceptible to treatment with 5-fluorouracil (5-FU) or cisplatin [41]. *SALL2* plays a pro-apoptotic role by regulating various transcriptional targets [19,44]. Furthermore, its levels increase in response to chemotherapeutic agents such as doxorubicin, which may explain the observed elevation following XAV939 treatment. Therefore, our findings highlight the importance of evaluating *SALL2*’s role in apoptotic mechanisms when using combination therapies involving XAV939 and chemotherapeutic agents such as 5-FU or oxaliplatin/cisplatin. Further research is needed to understand how the Wnt pathway regulates *SALL2* expression.

The regulation of *AXIN2* by *SALL2* may have significant implications for controlling the aberrant activation of the Wnt/β-catenin pathway in diseases. However, *AXIN2* regulation is complex and depends on several mechanisms, including epigenetics [55], post-transcriptional via ALKBH5-mediated m6A demethylation [56], transcriptional via CDX2 [57], or post-translational via tankyrase [58], among others. Thus, it is not surprising that, contrary to our main findings, some dataset analysis showed a negative association between *SALL2* and *AXIN2* mRNA levels in CRC. These results suggest that the effect of *SALL2* on *AXIN2* expression may be context-dependent.

*AXIN2* plays a crucial role in the phosphorylation and breakdown of β-catenin, forming a part of the destruction complex along with *AXIN1*. Despite their similarities, *AXIN1* and *AXIN2* are not interchangeable; *AXIN2* is vital for β-catenin degradation, as its suppression halts this process, whereas silencing *AXIN1* does not [59]. *AXIN2*, although typically a negative regulator of the Wnt/β-catenin pathway, can act as a promoter of oncogenesis by enhancing Snail1-driven epithelial−mesenchymal transition (EMT), which contributes to cancer cell invasion and metastasis [60], and has been detected overexpressed in CRC tissues, mainly in the cytoplasm of tumor epithelial cells [61]. In addition, *AXIN2* has important functions in the nucleus. It acts as a bridge between the transcription factors TCF and β-catenin, aiding in the reduction of target expression in the c-MYC pathway [62]. Nuclear import of AXIN is essential for relocating β-catenin to the cytoplasm. The shuttling of AXIN between the nucleus and cytoplasm enhances the export of β-catenin from the nucleus, facilitating its breakdown or interaction with E-cadherin in the cytoplasm, thereby modulating β-catenin signaling in the absence of Wnt signals. Future studies should clarify which AXIN functions depend on its regulation by *SALL2*.

Recent studies have identified *AXIN2* as a promising therapeutic target in the context of APC mutations [63,64,65,66]. Mechanistically, AXIN1/2 proteins are continuously degraded by tankyrase, an enzyme responsible for ADP-ribosylation [64]. Therefore, inhibiting tankyrase has been proposed to stabilize *AXIN2*, significantly suppressing the Wnt signaling pathway. Our research showed that *SALL2* is associated with an enhanced cell death response to XAV939 in colon cancer cells compared to CRC cells lacking *SALL2*. This finding suggests that *SALL2* enhances the effectiveness of the Wnt pathway inhibitor. However, it remains uncertain whether *SALL2* contributes to apoptosis in these cells independently of *AXIN2* in this context.

*SALL2* may serve as a potential biomarker for the suspicion of polyps, as there was a significant downregulation of *SALL2* in the early stages of CRC progression, such as adenoma, in our cohort. Although our study provided the first evidence for *SALL2* downregulation in CRC at the protein level, this observation was consistent with two previous studies showing a decrease in *SALL2* mRNA in this disease [12,46]. Together, these findings indicate the role of *SALL2* as a tumor suppressor in CRC. *SALL2* repression of the Wnt/β-catenin pathway via regulation of *AXIN2* and other WNT pathway regulators supports this notion. Moreover, survival analyses of our CRC patients suggested poorer outcomes for those with low *SALL2* expression. While earlier studies, such as Ma, T. et al., have linked *SALL2* expression to unfavorable outcomes in rectal cancer [46], our CRC dataset was primarily composed of colon cancer cases (44 out of 48 colorectal cancer instances), with only a small number of rectal cancer cases (*n* = 4), limiting any significant site-specific comparison. Nonetheless, the genetic context, or *SALL2* carrying some activating mutations, as demonstrated for p53, may shed light on this controversy. Further studies with additional patient cohorts, along with sequencing and transcriptome analyses, could help clarify the significance of *SALL2* expression in colon and rectal cancers.

## 4. Materials and Methods

### 4.1. Bioinformatics Analysis

We used the UALCAN database (http://ualcan.path.uab.edu/, accessed on 25 January 2025) to correlate tumor gene expression and survival. The screening conditions set in this study were as follows: “Gene: *SALL2*”, “Analysis Type: colon cancer vs. normal analysis”, and “Data Type: TCGA dataset”. We used the TIMER Database Analysis to analyze the differentially expressed genes in the context of CRC with *SALL2* mutations (http://cistrome.shinyapps.io/timer, accessed on 15 May 2025). This evaluation was conducted through the “Gene mutation” feature. In addition, we generated scatter plots of *SALL2* and *AXIN2* using publicly available databases and R2 software: Genomics Analysis and Visualization Platform (http://r2.amc.nl, accessed on 15 May 2025) using single-cell data [42,67]. Pearson’s correlation coefficients (r) and associated *p*-values (*p*) were calculated using the default HugoOnce algorithm and ANOVA statistical test.

### 4.2. Patients and Tissue Microarray Construction

The cohort was 130 paraffin-embedded human samples from colon cancer patients diagnosed at Guillermo Grant Benavente Hospital between 2018 and 2021, comprising adenomas (*n* = 40), CRC (*n* = 48; 44 colon and 4 rectal tumors), and healthy adjacent mucosa (*n* = 42). Formalin-fixed paraffin-embedded (FFPE) samples were obtained from patients, following a protocol approved by the Ethics Committee of the Universidad de Concepción and Guillermo Grant Benavente Hospital (Protocol #20-03-12, Concepción, Chile). The clinicopathological information obtained from each biopsy report was summarized in Supplementary Tables S1 and S2. Supplementary Table S2 focuses exclusively on the data from CRC patients (*n* = 48). A tissue microarray (TMA) was generated. Before TMA construction, tumor areas were identified and marked on hematoxylin/eosin-stained slices by specialized pathologists. TMA samples were prepared to comprise a core of 3.0 mm from each tumor. TMA sections of 4 μm (µm) were processed on microscope slides (FLEX IHC, Dako, Stockholm, Sweden) and dried in an oven for two hours at 60 °C.

### 4.3. Immunohistochemistry (IHC)

Protein expression in histological samples was analyzed in the TMA by IHC. Per the manufacturer’s instructions, we used 4 µm-thin sections from FFPE TMA on the EnVision™ FLEX, High pH (Dako Autostainer, Glostrup, Denmark). We used antibodies against *SALL2*, Ki-67, and β-Catenin, as detailed in Supplementary Table S3. For the previously characterized *SALL2* antibody [12,16,33,68], we standardized the optimal conditions to maximize *SALL2* antigen recovery in the colon samples. (Supplementary Figure S4a). To validate *SALL2* staining, *SALL2*-positive HEK293 cells were used as a positive control. Negative controls included the *SALL2*-positive HEK293 cells and normal colon tissue without the anti-*SALL2* antibody. Moreover, to confirm the specificity of the *SALL2* antibody, we utilized a previously described *SALL2* knockout (*SALL2*−/−) HEK293 cell model [16] (Supplementary Figure S4b).

### 4.4. Multiplexed Immunofluorescence (mIF)

Immunostaining was carried out as published [69] and each primary cocktail (or panel) was developed using validated antibodies [69,70]. Panels included Vimentin (clone V9, Thermo Scientific, Waltham, MA, USA), Cytokeratin (clone AE1/AE3, Invitrogen, Carlsbad, MA, USA), CD68 (clone PG-M1, Abcam, Cambridge, UK), CD8a (clone C8/144B, Dako, Glostrup, Denmark), and DAPI. All slides were scanned at a 20X magnification using an Aperio VERSA 200 microscope (Leica Biosystems, Vista, CA, USA). In the mIF analysis, segmentation of either the cytoplasm or nucleus was conducted as necessary using the open-source software QuPath v0.6.0. The quantification process relied on selecting regions of interest (ROIs) and co-localizing markers by employing automated thresholding.

### 4.5. IHC Analysis

The percentage of *SALL2*-positive cells was determined by counting 100 cells per image from 3 randomly selected high-power fields per sample at a 10× magnification using the cell counter tool of Image J software Version 1.54p. The analysis considered epithelial versus stromal cells and nuclear versus cytoplasmic localization. A positive staining was considered if *SALL2* was nuclear. Thus, the percentage of *SALL2*-positive nuclei in epithelium or stroma was obtained. Furthermore, *SALL2* staining intensity in the nucleus and cytoplasm was scored from 0 to 3 (no staining, weak staining, moderate staining, and strong staining). The β-catenin expression was evaluated as reported [71]. Evaluation of immunostaining was independently performed by two observers (A.Q. and C.D.), blinded to clinical data. The agreement between the two observers was >90%.

### 4.6. Agar-Cyto IHC

Cells (2 × 10^6^) were seeded in 6-well plates. Cells were fixed with methanol and pelleted by centrifugation for 10 min at 2000 rpm. The supernatant was removed, and the pellet was carefully resuspended in 1 mL of 2% liquid agarose at 65 °C (LE, analytical grade; Promega, Madison, WI, USA). The solidified agar was placed in a Tissue-Tek cassette (Sakura FineTek, Nagano, Japan) and embedded in paraffin using an automated tissue processor (Tissue-Tek VIP150; Sakura, Nagano, Japan) under standard conditions for surgical biopsies. Then, the IHC was performed in the agar-cyto.

### 4.7. Cell Culture and Lentiviral Transduction

HEK293 (ATCC, CRL-1573) and *SALL2KO* HEK293 [16] cells were cultured in DMEM (HyClone, Logan, UT, USA), with 10% fetal bovine serum (FBS, Biological Industries, Beit HaEmek, Israel) and 0.5% penicillin/streptomycin (Invitrogen, Carlsbad, MA, USA). CRC cell lines HT-29 (ATCC, HTB-38), SW480 (ATCC, CCL-228), SW480 *SALL2KO* [24], SW620 (ATCC, CCL-227), DLD-1 (ATCC, CCL-221), SW48 (ATCC, CCL-231), and HCT116 (a gift from Dr. Robert Warren, UCSF, USA) were cultured in RPMI-1640 (HyClone, Logan, UT, USA) supplemented with 10% FBS. Cells were cultured in a 37 °C and 5% CO_2_ atmosphere.

#### Generation of pCW57 Tet-On *FLAGSALL2E1A*

The pCW57 Tet-On FLAG-tagged *SALL2E1A* plasmid was generated by subcloning human FLAG-*SALL2E1A*, which was previously generated by ligating the human N-terminal FLAG-tagged *SALL2E1A* with the Exon 2 region of *SALL2E1*, conserved in both isoforms. The N-terminal exon 1 of *SALL2E1A* (ENST00000537235.2) tagged with FLAG was synthesized by GeneScript (http://www.genscript.com/, accessed on 15 May 2025) and amplified by PCR using the primers 5′-GCAGACCGGTATGGACTACAAAGACGATGA-3′ (forward) and 5′-TGGCAGCGACCAGGAAATGC-3′ (reverse). Exon 2 of *SALL2*, shared by both *SALL2* E1 and E1A isoforms, was digested from the pCW57 Tet-On FLAG-tagged human *SALL2E1* plasmid using BamHI to remove the N-terminal of the E1 isoform. The fragment containing only the Exon 2 was then ligated with the PCR-amplified FLAG-*SALL2E1A* fragment, generating the human FLAG-*SALL2E1A*, which was subsequently subcloned into the pCW57-MCS1-2A-MCS2 lentiviral vector using AgeI and AvrII restriction sites. The integrity of the full-length FLAG-tagged *SALL2E1A* coding sequence was confirmed by sequencing at Macrogen (Seoul, Republic of Korea, https://dna.macrogen.com/, accessed on 15 May 2025). The inducible expression of FLAG-tagged human *SALL2E1A* was confirmed by Western blot.

The reconstitution of *SALL2E1A* in the colorectal cancer cell lines HT-29, SW620, SW48, and SW480KO was as previously described for *SALL2KO* HEK293 cells [16]. For *SALL2E1A* induction, CRC or HEK293 cell lines were treated with doxycycline (1000 ng/mL) for 48 h before each experiment, and *SALL2* expression was confirmed by Western blot. The cell lines used in this study were regularly tested for mycoplasma using the EZ-PCR Mycoplasma Test Kit (Biological Industries, Beit HaEmek, Israel).

### 4.8. Immunofluorescence Staining

To evaluate the subcellular localization of β-catenin, HT29 and SW480 cell models were placed on coverslips and fixed using 4% paraformaldehyde for 30 min. Coverslips were rinsed with PBS, and cells were permeabilized with 0.2% Triton X-100 for 10 min. Following three PBS washes, cells were incubated with a β-catenin antibody (Supplementary Table S3) for 16 h. After being washed with PBS, cells were incubated with Alexa Fluor-488 conjugated secondary antibody (1:500 goat anti-mouse, Invitrogen) for 2 h and Hoechst 33342. After three times PBS washes, images were captured under confocal microscopy, LSM780 NLO Zeiss, in the Advanced Microscopy Center (CMA) at Universidad de Concepción. Nuclear segmentation utilized the DAPI channel to create a mask, which was then applied to the β-catenin green fluorescence channel to measure the nuclear fluorescence intensity. Image analysis was conducted using Fiji (ImageJ Version 1.54p) software to quantify the mean fluorescence intensity within the segmented nuclear areas.

### 4.9. Subcellular Fractionation

The nuclear/cytoplasmic fractionation was performed using an in-house protocol [72] with nuclear extraction buffer (10 mM HEPES, pH 7.9; 1.5 mM MgCl_2_; 10 mM KCl) and cytoplasmic extraction buffer (20 mM HEPES, pH 7.9; 25% (*v*/*v*) glycerol; 0.42 M NaCl; 1.5 mM MgCl_2_; 0.2 mM EDTA), and then, the lysate fractions were separated in an SDS/PAGE. HDAC1 and GAPDH were controls for nuclear and cytoplasmic fractions, respectively.

### 4.10. Western Blot Analysis

Cells were lysed in 25 mM Tris/HCl pH 7.5, 150 mM NaCl, 1% NP-40, 2 mM MgCl_2,_ and 5% glycerol, supplemented with protease and phosphatase inhibitor cocktails. Cell lysates fractionation and Western blot analysis were performed as previously (for primary antibodies and dilutions; see Supplementary Table S3) [16].

### 4.11. Real-Time Quantitative Reverse Transcription

Total RNA was extracted from cells with TRIzol reagent (Thermo Fisher Scientific, Waltham, MA, USA) according to the manufacturer’s instructions. RNA was treated with Turbo DNase (Thermo Fisher Scientific, Waltham, MA, USA). The retro-transcription was performed using MMLV-RT (Invitrogen, Carlsbad, MA, USA), 0.25 µg of Anchored Oligo(dT) 20 Primer (catalog 12577-011, Invitrogen, Carlsbad, MA, USA), and 2000 ng of RNA. qPCR was performed using KAPA SYBRG green (Kappa Biosystems, Wilmington, MA, USA). The forward and reverse primers are detailed in Supplementary Table S4.

### 4.12. Luciferase Reporter Gene

HEK293 *SALL2E1A* doxycycline-inducible cells (3 × 105) were seeded into 6-well plates and transfected with *AXIN2* 1000 pb promoter reporter by 24 h (a gift from Eric Fearon (Addgene plasmid # 25701) [35] or pGL3-Basic as a negative control and 0.125 μg of RSV-β-galactosidase (*GLB1*) used for internal normalization. Cells were harvested after 48 h and lysed using lysis buffer (Promega, Madison, WI, USA). Luciferase reporter gene assay was implemented using the Dual-Luciferase Reporter Assay System (Promega, Madison, WI, USA) as previously described [33].

### 4.13. Chromatin Immunoprecipitation Assay (ChIP Assay)

The ChIP assay was carried out as previously [33] with the following modifications: HEK293 FLAG-*SALL2E1A* doxycycline-inducible cells (2 × 10^6^ cells/100 mm plate) were seeded, and then, doxycycline (1μg/mL, for 48 h, Cayman Chemical #14422) was added to induce the expression of FLAG-*SALL2E1A*. Immunoprecipitations were carried out overnight at 4 °C using 1 μg anti-FLAG (M2, F1804, Sigma-Aldrich, St. Louis, MO, USA) or 5 μg normal mouse IgG antibodies and 40 μg of chromatin. DNA was analyzed by real-time PCR directed to *SALL2*-specific proximal regions of the *AXIN2* promoter. Primer sequences are detailed in Supplementary Table S3. In addition, a previously reported unrelated region (URR) of the *PMAIP1* promoter (−869/−756) was used as a negative control of *SALL2* binding [33]. All qPCRs were performed using a KAPA SYBR FAST kit (Kappa Biosystems, Wilmington, MA, USA) containing 1 μL of input and 3 μL of IP samples.

### 4.14. Statistical Analysis

Statistical analyses were performed using GraphPad Prism 8.0 software. Our results were presented as means ± standard deviation. Statistical significance was indicated with asterisks, and *p*-values were calculated using Chi-square, Student’s *t*-test, ANOVA, and post hoc analysis, wherein *, **, and *** represented *p* < 0.05, *p* < 0.01, and *p* < 0.001, respectively. Additional methods are available as Supplementary Materials accompanying the online article.

## 5. Conclusions

Our findings support a tumor suppressor role for *SALL2* in colorectal cancer, mediated in part through a previously unrecognized *SALL2*-dependent regulatory mechanism involving *AXIN2*, a key negative modulator of Wnt/β-catenin signaling. Loss of *SALL2* expression may contribute to hyperactivation of the Wnt/β-catenin pathway, thereby promoting tumor progression and influencing therapeutic responsiveness. Notably, *SALL2* enhances the pro-apoptotic effects of the Wnt pathway inhibition, suggesting its involvement in modulating treatment sensitivity. Future studies are warranted to further explore the role of the *SALL2*–*AXIN2* axis in response to standard chemotherapeutic agents such as 5-fluorouracil (5-FU) and to validate the clinical utility of *SALL2* as a prognostic and predictive biomarker in CRC.

## Data Availability

The original contributions presented in this study are included in the article/Supplementary Materials. Further inquiries can be directed to the corresponding author(s).

## Supplementary Materials

The following supporting information can be downloaded at: https://www.mdpi.com/article/10.3390/ijms26167896/s1.

## Abbreviations

The following abbreviations are used in this manuscript:

CRC—colorectal cancer; COAD—colon adenocarcinoma; READ—rectal adenocarcinoma; SALL—Spalt-like; *AXIN2*—axis inhibition protein 2/conductin; ChIP—chromatin immunoprecipitation; DMSO—dimethyl sulfoxide; EDTA—ethylene diamine tetra acetic acid; FBS—fetal bovine serum; GAPDH—glyceraldehyde-3-phosphate dehydrogenase; CK—casein kinase; GSK-3β— glycogen synthase kinase 3; TCF—T-cell factor/lymphoid enhancer factor; NLS—nuclear localization signal; PARP—Poli (ADP-Ribose) Polimerase 1; PFA—p-formaldehyde; LiCl—lithium chloride; PMAIP1—Phorbol-12-Myristate-13-Acetate-Induced Protein 1; SDS—Sodium Dodecyl Sulfate; FDA—food and drug administration; CMS—Consensus Molecular Subtype; SEM—Standard Error of the Media.

## References

[1] Bray F., Ferlay J., Soerjomataram I., Siegel R.L., Torre L.A., Jemal A. (2018). Global cancer statistics 2018: GLOBOCAN estimates of incidence and mortality worldwide for 36 cancers in 185 countries. CA Cancer J. Clin..

[2] Brierley J., Eycken E.V., Rous B.A., Giuliani M., O’Sullivan B. (2025). TNM Classification of Malignant Tumours.

[3] Lin C.H., Lin J.K., Chang S.C., Chang Y.H., Chang H.M., Liu J.H., Li L.H., Chen Y.T., Tsai S.F., Chen W.S. (2011). Molecular profile and copy number analysis of sporadic colorectal cancer in Taiwan. J. Biomed. Sci..

[4] Hu F., Wang J., Zhang M., Wang S., Zhao L., Yang H., Wu J., Cui B. (2021). Comprehensive Analysis of Subtype-Specific Molecular Characteristics of Colon Cancer: Specific Genes, Driver Genes, Signaling Pathways, and Immunotherapy Responses. Front. Cell. Dev. Biol..

[5] Menter D.G., Davis J.S., Broom B.M., Overman M.J., Morris J., Kopetz S. (2019). Back to the Colorectal Cancer Consensus Molecular Subtype Future. Curr. Gastroenterol. Rep..

[6] Thanki K., Nicholls M.E., Gomez G., Gajjar A., Senagore A.J., Rashidi L., Qiu S., Szabo C., Hellmich M.R., Chao C. (2017). Consensus Molecular Subtypes of Colorectal Cancer and their Clinical Implications. Int. Biol. Biomed. J..

[7] Liu J., Xiao Q., Xiao J., Niu C., Li Y., Zhang X., Zhou Z., Shu G., Yin G. (2022). Wnt/β-catenin signalling: Function, biological mechanisms, and therapeutic opportunities. Signal Transduct. Target. Ther..

[8] Hrckulak D., Kolar M., Strnad H., Korinek V. (2016). TCF/LEF transcription factors: An update from the internet resources. Cancers.

[9] Zhang Y., Wang X. (2020). Targeting the Wnt/β-catenin signaling pathway in cancer. J. Hematol. Oncol..

[10] Neiheisel A., Kaur M., Ma N., Havard P., Shenoy A.K. (2022). Wnt pathway modulators in cancer therapeutics: An update on completed and ongoing clinical trials. Int. J. Cancer.

[11] Hermosilla V.E., Hepp M.I., Escobar D., Farkas C., Riffo E.N., Castro A.F., Pincheira R. (2017). Developmental *SALL2* transcription factor: A new player in cancer. Carcinogenesis.

[12] Farkas C., Quiroz A., Alvarez C., Hermosilla V., Aylwin C.F., Lomniczi A., Castro A.F., Hepp M.I., Pincheira R. (2021). Characterization of *SALL2* Gene Isoforms and Targets Across Cell Types Reveals Highly Conserved Networks. Front. Genet..

[13] Sung C.K., Li D., Andrews E., Drapkin R., Benjamin T. (2013). Promoter methylation of the *SALL2* tumor suppressor gene in ovarian cancers. Mol. Oncol..

[14] Ye L., Lin C., Wang X., Li Q., Li Y., Wang M., Zhao Z., Wu X., Shi D., Xiao Y. (2019). Epigenetic silencing of SALL 2 confers tamoxifen resistance in breast cancer. EMBO J..

[15] Álvarez C., Quiroz A., Benítez-Riquelme D., Pincheira R., Riffo E., Castro A.F. (2021). SALL proteins; common and antagonistic roles in cancer. Cancers.

[16] Hermosilla V., Salgado G., Riffo E., Escobar D., Hepp M.I., Farkas C., Galindo M., Morín V., García-Robles M.A., Castro A.F. (2018). *SALL2* represses cyclins D1 and E1 expression and restrains G1/S cell cycle transition and cancer-related phenotypes. Mol. Oncol..

[17] Li D., Tian Y., Ma Y., Benjamin T. (2004). p150 (Sal2) is a p53-independent regulator if p21 (WAF/CIP). Mol. Cell. Biol..

[18] Wu Z., Cheng K., Shi L., Li Z., Negi H., Gao G., Kamle S., Li D. (2015). Sal-like protein 2 upregulates p16 expression through a proximal promoter element. Cancer Sci..

[19] Escobar D., Hepp M.I., Farkas C., Campos T., Sodir N.M., Morales M., Álvarez C.I., Swigart L., Evan G.I., Gutiérrez J.L. (2015). Sall2 is required for proapoptotic Noxa expression and genotoxic stress-induced apoptosis by doxorubicin. Cell Death Dis..

[20] Suvà M.L., Rheinbay E., Gillespie S.M., Patel A.P., Wakimoto H., Rabkin S.D., Riggi N., Chi A.S., Cahill D.P., Nahed B.V. (2014). Reconstructing and reprogramming the tumor-propagating potential of glioblastoma stem-like cells. Cell.

[21] Onai T., Sasai N., Matsui M., Sasai Y. (2004). Xenopus XsalF: Anterior neuroectodermal specification by attenuating cellular responsiveness to Wnt signaling. Dev. Cell.

[22] Clevers H., Nusse R. (2012). Wnt/β-catenin signaling and disease. Cell.

[23] Gao C., Xiao G., Hu J. (2014). Regulation of Wnt/β-catenin signaling by posttranslational modifications. Cell Biosci..

[24] Hermosilla V.E., Gyenis L., Rabalski A.J., Armijo M.E., Sepúlveda P., Duprat F., Benítez-Riquelme D., Fuentes-Villalobos F., Quiroz A., Hepp M.I. (2024). Casein kinase 2 phosphorylates and induces the *SALL2* tumor suppressor degradation in colon cancer cells. Cell Death Dis..

[25] Binnerts M.E., Kim K.-A., Bright J.M., Patel S.M., Tran K., Zhou M., Leung J.M., Liu Y., Lomas W.E., Dixon M. (2007). R-Spondin1 regulates Wnt signaling by inhibiting internalization of LRP6. Proc. Natl. Acad. Sci. USA.

[26] Upadhyay G., Goessling W., North T.E., Xavier R., Zon L.I., Yajnik V. (2008). Molecular association between β-catenin degradation complex and Rac guanine exchange factor DOCK4 is essential for Wnt/β-catenin signaling. Oncogene.

[27] Bernkopf D.B., Brückner M., Hadjihannas M.V., Behrens J. (2019). An aggregon in conductin/axin2 regulates Wnt/β-catenin signaling and holds potential for cancer therapy. Nat. Commun..

[28] Jho E., Zhang T., Domon C., Joo C.-K., Freund J.-N., Costantini F. (2002). Wnt/β-Catenin/Tcf Signaling Induces the Transcription of Axin2, a Negative Regulator of the Signaling Pathway. Mol. Cell. Biol..

[29] Galli C., Piemontese M., Lumetti S., Manfredi E., Macaluso G.M., Passeri G. (2013). GSK3b-inhibitor lithium chloride enhances activation of Wnt canonical signaling and osteoblast differentiation on hydrophilic titanium surfaces. Clin. Oral Implant. Res..

[30] Wang B., Khan S., Wang P., Wang X., Liu Y., Chen J., Tu X. (2022). A Highly Selective GSK-3β Inhibitor CHIR99021 Promotes Osteogenesis by Activating Canonical and Autophagy-Mediated Wnt Signaling. Front. Endocrinol..

[31] Makola R.T., Kgaladi J., More G.K., van Vuren P.J., Paweska J.T., Matsebatlela T.M. (2021). Lithium inhibits NF-κB nuclear translocation and modulate inflammation profiles in Rift valley fever virus-infected Raw 264.7 macrophages. Virol. J..

[32] Kim J.Y., Park H.H., Yong T.-S., Jeon S.-H. (2021). Lithium chloride inhibits the migration and invasion of osteosarcoma cells by blocking nuclear translocation of phospho-Erk. Biochem. Biophys. Res. Commun..

[33] Riffo E., Palma M., Hepp M.I., Benítez-Riquelme D., Torres V.A., Castro A.F., Pincheira R. (2022). The Sall2 transcription factor promotes cell migration regulating focal adhesion turnover and integrin β1 expression. Front. Cell Dev. Biol..

[34] Gu H., Li D., Sung C.K., Yim H., Troke P., Benjamin T. (2011). DNA-binding and regulatory properties of the transcription factor and putative tumor suppressor p150Sal2. Biochim. Biophys. Acta Gene Regul. Mech..

[35] Leung J.Y., Kolligs F.T., Wu R., Zhai Y., Kuick R., Hanash S., Cho K.R., Fearon E.R. (2002). Activation of *AXIN2* expression by β-catenin-T cell factor: A feedback repressor pathway regulating Wnt signaling. J. Biol. Chem..

[36] Karantzali E., Schulz H., Hummel O., Hubner N., Hatzopoulos A.K., Kretsovali A. (2008). Histone deacetylase inhibition accelerates the early events of stem cell differentiation: Transcriptomic and epigenetic analysis. Genome Biol..

[37] Pasini D., Malatesta M., Jung H.R., Walfridsson J., Willer A., Olsson L., Skotte J., Wutz A., Porse B., Jensen O.N. (2010). Characterization of an antagonistic switch between histone H3 lysine 27 methylation and acetylation in the transcriptional regulation of Polycomb group target genes. Nucleic Acids Res..

[38] Bao R., Christova T., Song S., Angers S., Yan X., Attisano L. (2012). Inhibition of Tankyrases Induces Axin Stabilization and Blocks Wnt Signalling in Breast Cancer Cells. PLoS ONE.

[39] Huang S.M.A., Mishina Y.M., Liu S., Cheung A., Stegmeier F., Michaud G.A., Charlat O., Wiellette E., Zhang Y., Wiessner S. (2009). Tankyrase inhibition stabilizes axin and antagonizes Wnt signalling. Nature.

[40] Fu F., Deng Q., Li R., Wang D., Yu Q.-X., Yang X., Lei T.-Y., Han J., Pan M., Zhen L. (2020). *AXIN2* gene silencing reduces apoptosis through regulating mitochondria-associated apoptosis signaling pathway and enhances proliferation of ESCs by modulating Wnt/β-catenin signaling pathway. Eur. Rev. Med. Pharmacol. Sci..

[41] Wu X., Luo F., Li J., Zhong X., Liu K. (2016). Tankyrase 1 inhibitior XAV939 increases chemosensitivity in colon cancer cell lines via inhibition of the Wnt signaling pathway. Int. J. Oncol..

[42] Qin X., Rodriguez F.C., Sufi J., Vlckova P., Claus J., Tape C.J. (2023). An oncogenic phenoscape of colonic stem cell polarization. Cell.

[43] Cerami E., Gao J., Dogrusoz U., Gross B.E., Sumer S.O., Arman B., Jacobsen A., Byrne C.J., Heuer M.L., Larsson E. (2012). The cBio Cancer Genomics Portal: An Open Platform for Exploring Multidimensional Cancer Genomics Data. Cancer Discov..

[44] Sung C.K., Yim H., Gu H., Li D., Andrews E., Duraisamy S., Li C., Drapkin R., Benjamin T. (2012). The Polyoma Virus Large T Binding Protein p150 Is a Transcriptional Repressor of c-MYC. PLoS ONE.

[45] Pincheira R., Baerwald M., Dunbar J.D., Donner D.B. (2009). Sall2 is a novel p75NTR-interacting protein that links NGF signalling to cell cycle progression and neurite outgrowth. EMBO J..

[46] Ma T., Shi S., Jiang H., Chen X., Xu D., Ding X., Zhang H., Xi Y. (2021). A pan-cancer study of spalt-like transcription factors 1/2/3/4 as therapeutic targets. Arch. Biochem. Biophys..

[47] Souza L.C.d.M.e., Faletti A., Veríssimo C.P., Stelling M.P., Borges H.L. (2022). p53 Signaling on Microenvironment and Its Contribution to Tissue Chemoresistance. Membranes.

[48] Bian J., Dannappel M., Wan C., Firestein R. (2020). Transcriptional Regulation of Wnt/β-Catenin Pathway in Colorectal Cancer. Cells.

[49] Sato A., Kishida S., Tanaka T., Kikuchi A., Kodama T., Asashima M., Nishinakamura R. (2004). Sall1, a causative gene for Townes–Brocks syndrome, enhances the canonical Wnt signaling by localizing to heterochromatin. Biochem. Biophys. Res. Commun..

[50] Chen M., Li L., Zheng P.S. (2019). SALL4 promotes the tumorigenicity of cervical cancer cells through activation of the Wnt/β-catenin pathway via CTNNB1. Cancer Sci..

[51] Ma C., Wang F., Han B., Zhong X., Si F., Ye J., Hsueh E.C., Robbins L., Kiefer S.M., Zhang Y. (2018). SALL1 functions as a tumor suppressor in breast cancer by regulating cancer cell senescence and metastasis through the NuRD complex. Mol. Cancer.

[52] Zhang W., Hu Y., Zhang W., Yi K., Xu X., Chen Z. (2022). The Invasion and Metastasis of Colon Adenocarcinoma (COAD) Induced by SALL4. J. Immunol. Res..

[53] Bernkopf D.B., Hadjihannas M.V., Behrens J. (2014). Negative feedback regulation of the Wnt pathway by conductin/*AXIN2* involves insensitivity to upstream signalling. J. Cell Sci..

[54] Kelberman D., Islam L., Lakowski J., Bacchelli C., Chanudet E., Lescai F., Patel A., Stupka E., Buck A., Wolf S. (2014). Mutation of *SALL2* Causes Recessive Ocular Coloboma in Humans and Mice. Hum. Mol. Genet..

[55] Koinuma K., Yamashita Y., Liu W., Hatanaka H., Kurashina K., Wada T., Takada S., Kaneda R., Choi Y.L., Fujiwara S.-I. (2006). Epigenetic silencing of *AXIN2* in colorectal carcinoma with microsatellite instability. Oncogene.

[56] Zhai J., Chen H., Wong C.C., Peng Y., Gou H., Zhang J., Pan Y., Chen D., Lin Y., Wang S. (2023). ALKBH5 Drives Immune Suppression Via Targeting *AXIN2* to Promote Colorectal Cancer and Is a Target for Boosting Immunotherapy. Gastroenterology.

[57] Yu J., Liu D., Sun X., Yang K., Yao J., Cheng C., Wang C., Zheng J. (2019). CDX2 inhibits the proliferation and tumor formation of colon cancer cells by suppressing Wnt/β-catenin signaling via transactivation of GSK-3β and Axin2 expression. Cell Death Dis..

[58] Jang M.-K., Mashima T., Seimiya H. (2020). Tankyrase Inhibitors Target Colorectal Cancer Stem Cells via AXIN-Dependent Downregulation of c-KIT Tyrosine Kinase. Mol. Cancer Ther..

[59] Wang W., Liu P., Lavrijsen M., Li S., Zhang R., Li S., van de Geer W.S., van de Werken H.J.G., Peppelenbosch M.P., Smits R. (2021). Evaluation of *AXIN1* and *AXIN2* as targets of tankyrase inhibition in hepatocellular carcinoma cell lines. Sci. Rep..

[60] Wu Z.-Q., Brabletz T., Fearon E., Willis A.L., Hu C.Y., Li X.-Y., Weiss S.J. (2012). Canonical Wnt suppressor, Axin2, promotes colon carcinoma oncogenic activity. Proc. Natl. Acad. Sci. USA.

[61] Schaal U., Grenz S., Merkel S., Rau T.T., Hadjihannas M.V., Kremmer E., Chudasama P., Croner R.S., Behrens J., Stürzl M. (2013). Expression and localization of axin 2 in colorectal carcinoma and its clinical implication. Int. J. Color. Dis..

[62] Rennoll S.A., Konsavage W.M., Yochum G.S. (2014). Nuclear *AXIN2* represses MYC gene expression. Biochem. Biophys. Res. Commun..

[63] Stakheev D., Taborska P., Strizova Z., Podrazil M., Bartunkova J., Smrz D. (2019). The WNT/β-catenin signaling inhibitor XAV939 enhances the elimination of LNCaP and PC-3 prostate cancer cells by prostate cancer patient lymphocytes in vitro. Sci. Rep..

[64] Thorvaldsen T.E., Pedersen N.M., Wenzel E.M., Stenmark H. (2017). Differential roles of *AXIN1* and *AXIN2* in tankyrase inhibitor-induced formation of degradasomes and β-catenin degradation. PLoS ONE.

[65] Waaler J., Machon O., Tumova L., Dinh H., Korinek V., Wilson S.R., Paulsen J.E., Pedersen N.M., Eide T.J., Machonova O. (2012). A novel tankyrase inhibitor decreases canonical Wnt signaling in colon carcinoma cells and reduces tumor growth in conditional APC mutant mice. Cancer Res..

[66] Wu D., Talbot C.C., Liu Q., Jing Z.-C., Damico R.L., Tuder R., Barnes K.C., Hassoun P.M., Gao L. (2016). Identifying microRNAs targeting Wnt/β-catenin pathway in end-stage idiopathic pulmonary arterial hypertension. J. Mol. Med..

[67] Joanito I., Wirapati P., Zhao N., Nawaz Z., Yeo G., Lee F., Eng C.L.P., Macalinao D.C., Kahraman M., Srinivasan H. (2022). Single-cell and bulk transcriptome sequencing identifies two epithelial tumor cell states and refines the consensus molecular classification of colorectal cancer. Nat. Genet..

[68] Hepp M.I., Escobar D., Farkas C., Hermosilla V.E., Álvarez C., Amigo R., Gutiérrez J.L., Castro A.F., Pincheira R. (2018). A Trichostatin A (TSA)/Sp1-mediated mechanism for the regulation of *SALL2* tumor suppressor in Jurkat T cells. Biochim. Biophys. Acta Gene Regul. Mech..

[69] Cereceda K., Bravo N., Jorquera R., González-Stegmaier R., Villarroel-Espíndola F. (2022). Simultaneous and Spatially-Resolved Analysis of T-Lymphocytes, Macrophages and PD-L1 Immune Checkpoint in Rare Cancers. Cancers.

[70] Martinez-Morilla S., Villarroel-Espindola F., Wong P.F., Toki M.I., Aung T.N., Pelekanou V., Bourke-Martin B., Schalper K.A., Kluger H.M., Rimm D.L. (2021). Biomarker discovery in patients with immunotherapy-treated melanoma with imaging mass cytometry. Clin. Cancer Res..

[71] Galera-Ruiz H., Ríos M.J., González-Cámpora R., de Miguel M., Carmona M.I., Moreno A.M., Galera-Davidson H. (2011). The cadherin–catenin complex in nasopharyngeal carcinoma. Eur. Arch. Oto-Rhino-Laryngol..

[72] Palma M., Riffo E.N., Suganuma T., Washburn M.P., Workman J.L., Pincheira R., Castro A.F. (2019). Identification of a nuclear localization signal and importin beta members mediating NUAK1 nuclear import inhibited by oxidative stress. J. Cell Biochem..

