# Supplementary material for: SALL2-Mediated Suppression of WNT Signaling Through Transcriptional Control of AXIN2 in Colorectal Cancer Cells
Source: Int J Mol Sci. 2025 Aug 15;26(16):7896. doi: 10.3390/ijms26167896 (PMC12387037; doi:10.3390/ijms26167896)
Supplement: Supplementary file 1 [file ijms-26-07896-s001.zip › ijms-3735553-supplementary.pdf]

SALL2-Mediated Suppression of WNT Signaling Through Transcriptional Control of AXIN2 in Colorectal Cancer Cells

Aracelly Quiroz [1,2,3], Emilia Escalona [4], Carlos Farkas [5], Diego Benítez-Riquelme [1,2], Paulina Sepúlveda [1,2], Mario Palma [1,2,†], Paula Medina [1,2], Carolina Delgado [3], Matías I. Hepp [5], Franz Villarroel-Espindola [6], Ariel F. Castro [1,2,*] and Roxana Pincheira [1,2,*]

1 Departamento de Bioquímica y Biología Molecular, Facultad de Ciencias Biológicas, Universidad de Concepción,4070409 Concepción, Chile; aracellyquiroz@udec.cl (A.Q.); diegobenitez@udec.cl (D.B.-R.); psepulv.ps@gmail.com (P.S.); mpalma@hsph.harvard.edu (M.P.); pmedina2018@udec.cl (P.M.)

2 Laboratorio de Transducción de Señales y Cáncer, Facultad de Ciencias Biológicas, Universidad de Concepción, Concepción, Chile

3 Departamento de Especialidades, Facultad de Medicina, Universidad de Concepción, Concepción, Chile; carolinadelgado@udec.cl

4 MARLab, Instituto de Ciencias Biomédicas, Facultad de Ciencias de la Salud, Universidad Autónoma de Chile, Talca, Chile; emilia.escalona@uautonoma.cl

5 Laboratorio de Investigación en Ciencias Biomédicas, Departamento de Ciencias Básicas y Morfología, Facultad de Medicina, Universidad Católica de la Santísima Concepción, Concepción, Chile; cfarkas@ucsc.cl (C.F.); mhepp@ucsc.cl (M.I.H.)

6 Translational Medicine Laboratory, Fundación Arturo López Pérez Cancer Center, Santiago, Chile; franz.villarroel@falp.org

* Correspondence: arcastro@udec.cl (A.F.C.); ropincheira@udec.cl (R.P.); Tel.: +56-412203794 (A.F.C.); +56-412203815 (R.P.)

† Current address: Department of Molecular Metabolism, Harvard T.H. Chan School of Public Health, Boston, MA 02115, USA.

Aracelly Quiroz: https://orcid.org/0009-0000-5221-4762

Emilia Escalona: https://orcid.org/0009-0005-1196-1282

Carlos Farkas: https://orcid.org/0000-0002-6245-2622

Diego Benítez: https://orcid.org/0000-0001-5470-5792

Mario Palma: https://orcid.org/0009-0007-9456-2096

Matías Hepp: https://orcid.org/0000-0003-0993-8794

Franz Villarroel: https://orcid.org/0000-0003-0080-2444

Ariel Castro: https://orcid.org/0000-0001-5583-4879

Roxana Pincheira: https://orcid.org/0000-0003-4277-5245

Corresponding author: Roxana Pincheira, Departamento de Bioquímica y Biología Molecular, Facultad Cs. Biológicas. Universidad de Concepción. Chile. Phone: 56-412203815; E-mail: ropincheira@udec.cl. Ariel Castro. Departamento de Bioquímica y Biología Molecular, Facultad Cs. Biológicas. Universidad de Concepción. Chile. Phone: 56-412203794; E-mail: arcastro@udec.cl.

Running Title: The SALL2/AXIN2 axis-dependent regulation of the Wnt pathway

Keywords: SALL2, AXIN2, Wnt-β catenin, CHIR99021, XAV393, CRC

Supplementary figures:

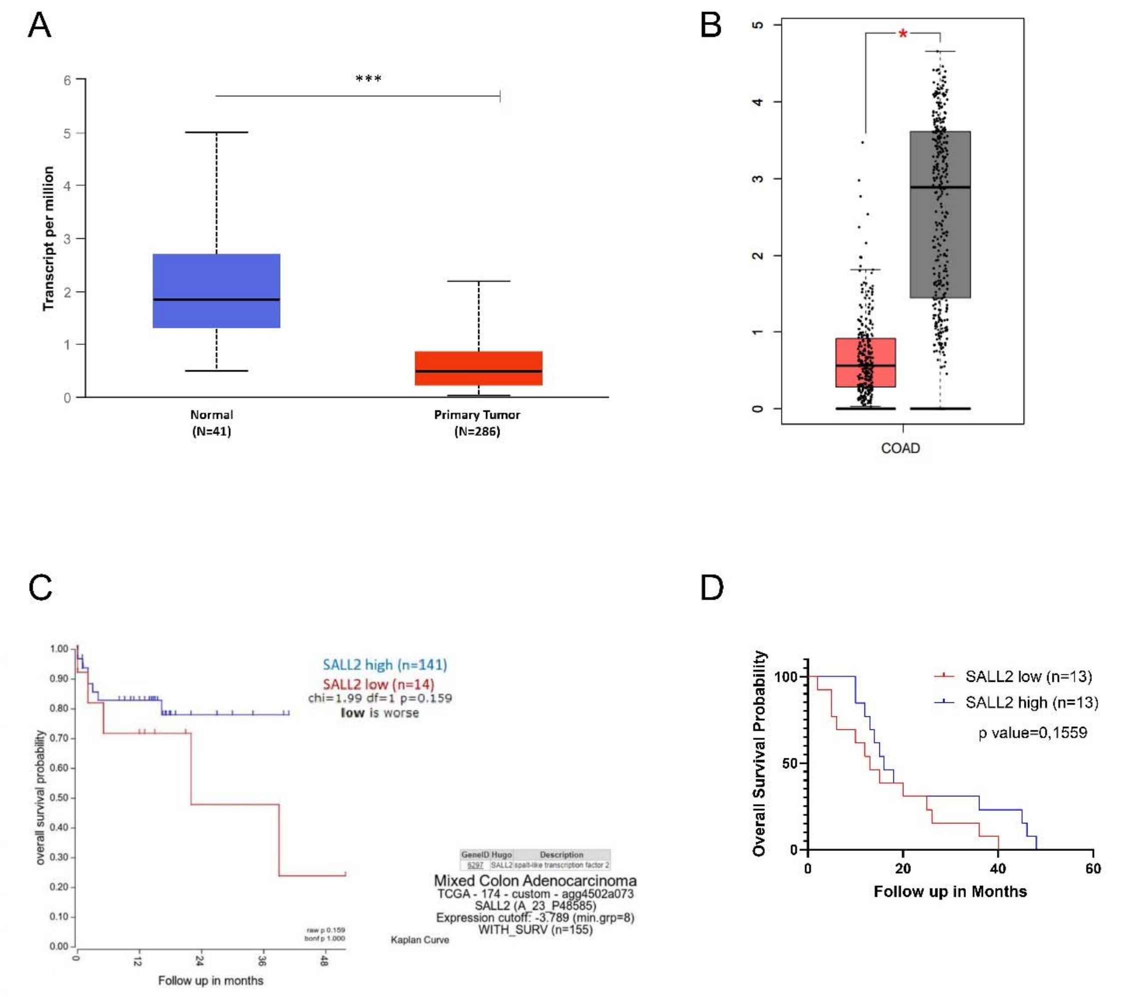

Supplementary Figure S1: ***SALL2*** **expression is downregulated in CRC and related to overall survival A)** *SALL2* mRNA expression levels in TCGA colon tumors (n = 286, blue bar) and matching normal tissue (n = 41, red bar) from UALCAN database (*p=7,5E-05*) **B)** The *SALL2* mRNA expression levels in TCGA normal (n=349) and GTEx cancer (n=275) data from GEPIA database (*p<0.05*). In red cancer tissue and grey normal tissue. **C)** Kaplan–Meier analysis of Overall survival (OS) curves for patients with high *SALL2* (n = 141) or low *SALL2* (n = 14) from TCGA Mixed colon adenocarcinoma cohort using R2 Genomics: dataset (https://hgserver1.amc.nl/cgi-bin/r2/main.cgi), ***P < 0.001, *P < 0.05. **D)** Kaplan–Meier analysis of OS curves for patients with high *SALL2* (n = 13) or low *SALL2* (n = 13) who died from cancer in our local study.

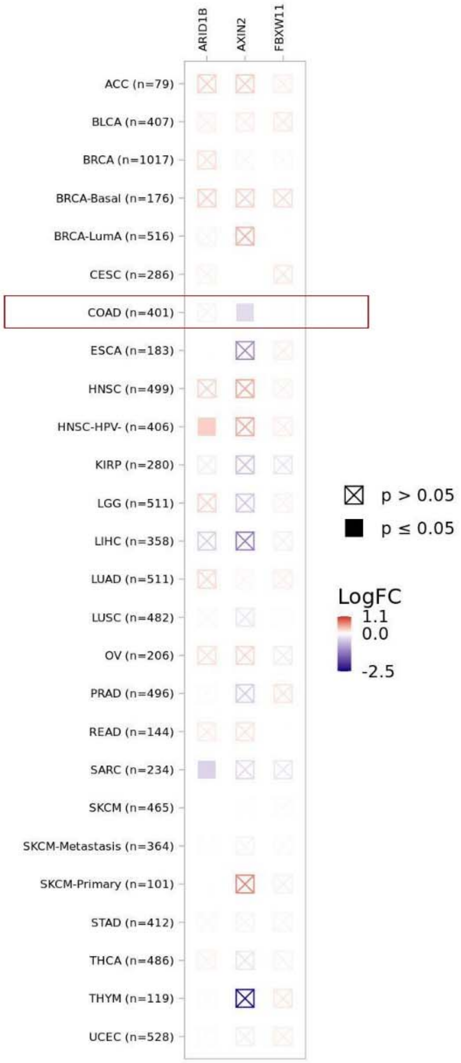

Supplementary Figure S2: *AXIN2* mRNA is downregulated in Colon Adenocarcinoma (COAD) tissues with *SALL2* genetic alterations. The functional heatmap table generated by the 'Gene Module' in the TIMER 2.0 platform shows the association between *SALL2*-mutated expression and Wnt pathway-related genes *ARID1B*, *AXIN2*, and *FBXW11* across TCGA cancer types. The red indicates a statistically significant positive association, and the blue indicates a statistically significant negative association. Gray denotes a non-significant result.

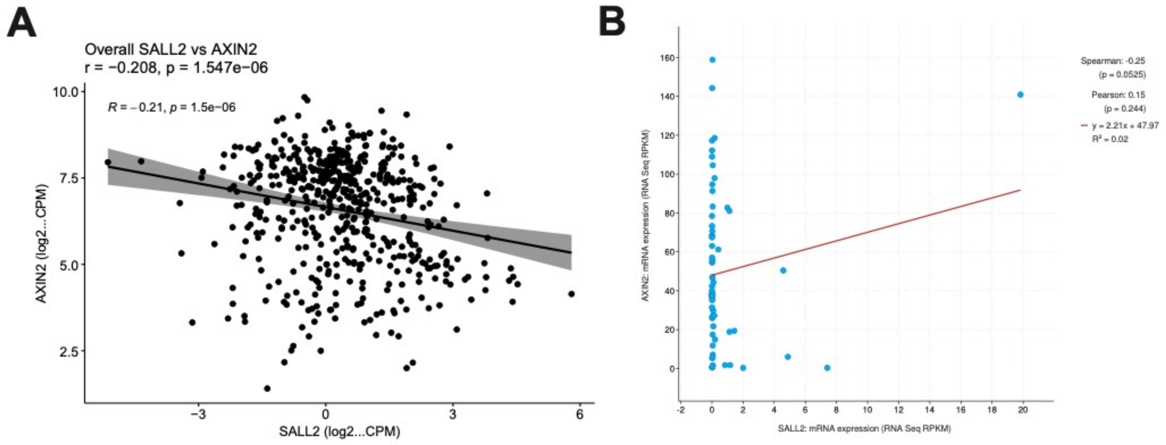

Supplementary Figure S3: Correlation between *SALL2* and *AXIN2* genes across bulk RNA sequencing datasets. A) Primary colorectal tumors (TCGA COAD). Scatter plot of log_2 CPM–normalized RNA seq counts for *SALL2* (x axis) and *AXIN2* (y axis) across all evaluable TCGA COAD samples (STAR Counts workflow). A best-fit linear regression (black line ± 95 % CI, grey band) illustrates a modest but significant inverse association (Pearson $r = –0.21$, $P = 1.5 \times 10^{-6}$; Spearman $\varrho = –0.21$). **B).** Correlation of *SALL2* and *AXIN2* mRNA expression (RNA seq RPKM) in 60 colorectal cancer cell lines, visualized with the cBioPortal web server (dataset: Cancer Cell Line Encyclopedia – Broad 2019). Although Pearson's correlation is weak and not significant ($r = 0.15$, $P = 0.244$), Spearman correlation shows a trend towards a negative relationship ($\varrho = –0.25$, $P = 0.053$).

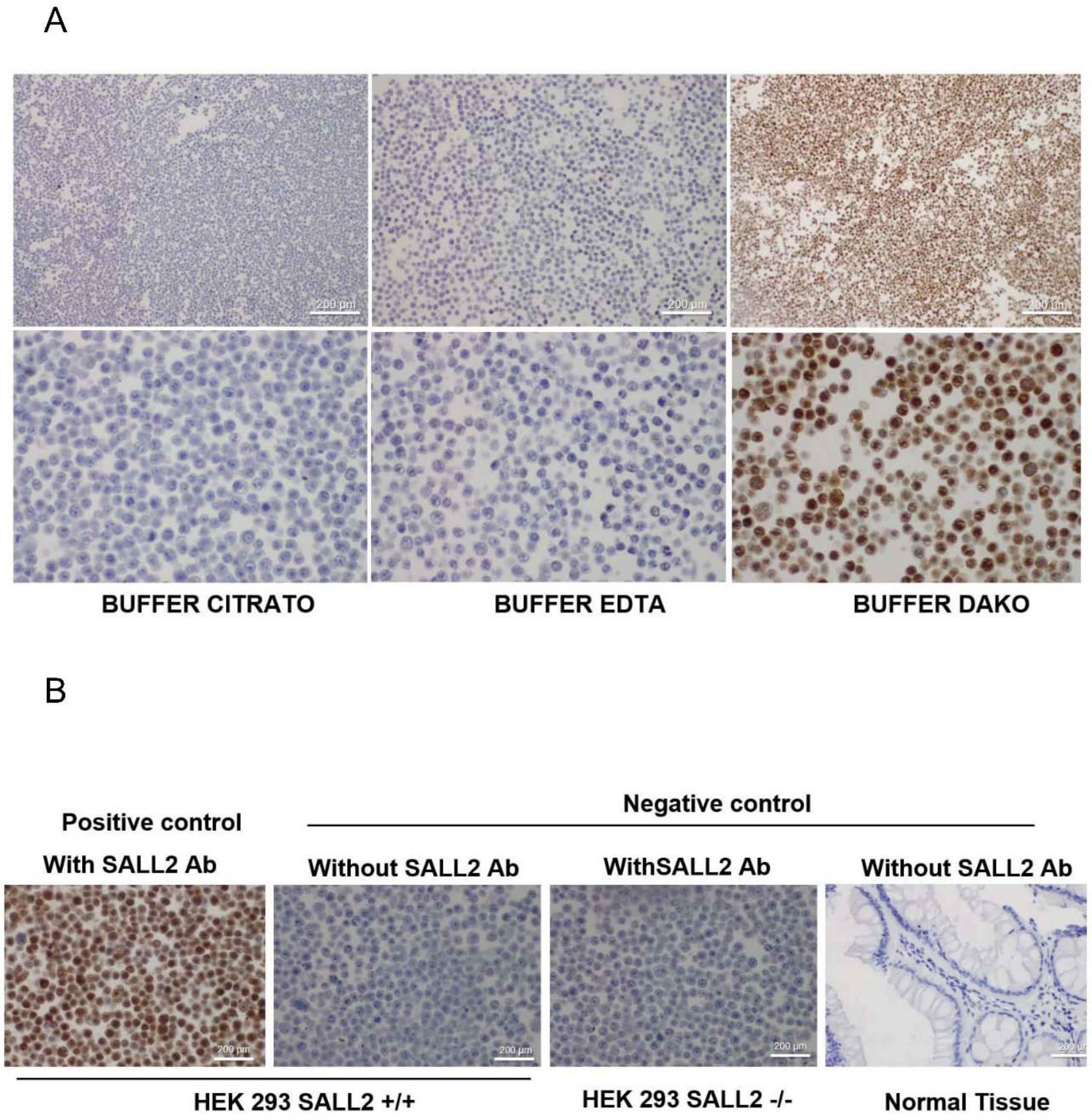

Supplementary Figure S4: Standardization of IHC staining and antibody specificity. A) SALL2 +/+ HEK293 cells were processed based on the AgarCyto cell block preparation for IHC analysis. The efficacy of 3 different antigen retrieval buffers was tested (Citrate, EDTA, and DAKO), and the DAKO buffer was the most effective. Representative SALL2 IHC are shown (1:50 dilution). **B)** Representative SALL2 IHC images in SALL2 +/+ HEK293 cells (1:50, positive control) and negative controls: SALL2 +/+ HEK 293 cells without SALL2 antibody, SALL2 $^{-/-}$ HEK293 cells with the SALL2 antibody (1:50 dilution), and normal colon tissue without the SALL2 antibody.

SUPPLEMENTARY TABLES:

Supplementary Table S1: Clinical and Demographic Patient Information.

**Diagnosis**	**n**	**Women**	**Men**	**Age at diagnosis**
**Normal**	42	20/42 (47,6%)	22/42 (52,4%)	70
**Adenoma**	40	19/40 (47,5%)	21/40 (52,5%)	63,5
**CRC**	48	27/48 (56%)	21/48 (43,7%)	69,6
**Total**	**130**	**66**	**64**	**67,7**

Supplementary Table S2: CRC Patients Clinical characteristics

**Clinical characteristics**		SALL2 Negative		SALL2 Positive		p-value
		N	%	N	%	
**Sex**	Female	18	66,67	9	33,33	0,2357
	Male	16	76,19	5	23,81	
**Age**	> 60	26	83,87	5	16,13	0,0036
	< 60	8	47,06	9	52,94	
**Tumor Size**	< 5 cm	8	57,14	6	42,86	0,0903
	> 5 cm	26	76,47	8	23,53	
**Tumor Localization**	Colon	32	72,72%	12	27,27%	0,5695
	Rectum	2	50%	2	50%	
**T**	T1-T2	4	50,00	4	50,00	0,0778
	T3-T4	30	75,00	10	25,00	
**N**	N0	18	69,23	8	30,77	0,9503
	N1	6	75,00	2	25,00	
	N2	10	71,43	4	28,57	
**Invasion**	Mucosa	2	50,00	2	50,00	0,5649
	Submucosa	1	33,33	2	66,67	
	Serosa	31	75,61	10	24,39	
**Microsatelital inestability**	Positive	33	73,33	12	26,67	0,0700
	Negative	1	33,33	2	66,67	
**β-catenin on the migratory front**	Positive	24	80,00	6	20,00	0,0356
	Negative	10	55,56	8	44,44	
**Linfovascular Permeation**	Positive	19	76,00	6	24,00	0,2058
	Negative	15	65,22	8	34,78	
**Ki67**	Negative	22	75,86	7	24,13	0,2654
	Positive	12	63,15	7	36,84	

Supplementary Table S3: Primary antibodies.

Antibody	Clone	Source	Method	Dilution	Source
**SALL2**	HPA004162	SIGMA	WB	1:500	Rabbit
			IHQ	1:50	
**ARID1B**	(KMN1): sc- 32762	Santa Cruz	WB	1:350	Mouse
**ARID1A**	(C-7): sc- 373784	Santa Cruz	WB	1:350	Mouse
**FBXW7**	(A-4): sc- 518093	Santa Cruz	WB	1:350	Mouse
**FBXW11**	(C-6): sc- 390629	Santa Cruz	WB	1:350	Mouse
**LEF1**	(B-6): sc-374522.	Santa Cruz	WB	1:500	Mouse
**DAAM2**	(E-1): sc-515129	Santa Cruz	WB	1:500	Mouse
**WNT7b**	(H-8): sc-365459	Santa Cruz	WB	1:350	Mouse
**WNT3a**	**(3A6): sc-136163**	Santa Cruz	WB	1:350	Mouse
**AXIN2**	(C-6): sc-25302.	Santa Cruz	WB	1:500	Mouse
		Santa Cruz	IF	1:25	
**β-catenin**	(e-5): sc-7963	Santa Cruz	WB	1:100	Mouse
**β-Actin**	(C4): sc-47778	Santa Cruz	WB	1:20000	Mouse
**GAPDH**	(e-5): sc-7963	Santa Cruz	WB	1:20000	Mouse

**HDAC 1**	(C-19): sc-6298	Santa Cruz	WB	1:500	Goat
**Flag**	Anti-Flag M2, clone M2	SIGMA	WB	1:5000	Mouse
**Cleaved PARP**	(194C1439): sc-56196	Santa Cruz	WB	1:500	Mouse
**Cleaved caspase 3**	(31A1067): sc-56053	Santa Cruz	WB	1:500	Mouse

Supplementary Table 4: Primer sequences for qRT-PCR and ChIP

Partidores	Forward primer (5′-3′)	Reverse primer (5′-3′)
AXIN2	CCCATGCCTGTCTCTTCCAA	ATCCACGCATTTCTCCCTCT
ACTIN	GATGAGATTGGCATGGCTTT	CACCTTCACCGTTCCAGTTT
PPIB	AACGCAGGCAAAGACACCAA CG	TCTGTCTTGGTGCTCTCCACCT
AXIN2 (-1420/-1410/-1405/-1400)	CTGTTGCATTCCAGAGCCG	CCTTAAAGGGACAGCGCC
AXIN2 (-784)	CGCCTTTGAAGTGCACAGTT	TTATGCAAAAGATCCGAGCGG
AXIN2 (-112/-108)	TTATGGATGTGAGTCGGCGT	GAGGGGAAAAGGGGAGGG
URR (ChIP)	TGAAGCGGCTCTCAGTAACC	AGCTACCTGGGAACGTGAAA
